# Locality Estimates for Complex Time Evolution in 1D

**DOI:** 10.1007/s00220-022-04573-w

**Published:** 2023-01-03

**Authors:** David Pérez-García, Antonio Pérez-Hernández

**Affiliations:** 1 Instituto de Ciencias Matemáticas, 28049 Madrid, Spain; 2 Departamento de Análisis Matemático y Matemática Aplicada, Universidad Complutense de Madrid, 28040 Madrid, Spain; 3 Departamento de Matemática Aplicada I, Universidad Nacional de Educación a Distancia, 28040 Madrid, Spain

## Abstract

It is a generalized belief that there are no thermal phase transitions in short range 1D quantum systems. However, the only known case for which this is rigorously proven is for the particular case of *finite range* translationally invariant interactions. The proof was obtained by Araki in his seminal paper of 1969 as a consequence of pioneering locality estimates for the time-evolution operator that allowed him to prove its analyticity on the whole complex plane, when applied to a local observable. However, as for now there is no mathematical proof of the absence of 1D thermal phase transitions if one allows exponential tails in the interactions. In this work we extend Araki’s result to include exponential (or faster) tails. Our main result is the analyticity of the time-evolution operator applied on a local observable on a suitable strip around the real line. As a consequence we obtain that thermal states in 1D exhibit exponential decay of correlations above a threshold temperature that decays to zero with the exponent of the interaction decay, recovering Araki’s result as a particular case. Our result however still leaves open the possibility of 1D thermal short range phase transitions. We conclude with an application of our result to the spectral gap problem for Projected Entangled Pair States (PEPS) on 2D lattices, via the holographic duality due to Cirac et al.

## Introduction

Lieb–Robinson bounds [28] are described as a non-relativistic counterpart to the finite-speed limit for the propagation of signals or perturbations in certain quantum systems. They formally establish that the dynamical evolution of a local observable under a local Hamiltonian has an approximately bounded support that grows linearly in the time variable and its velocity depends on the interaction and the underlying metric structure. For the last 15 years, these locality bounds have been sharpened and extended, motivated by a wide range of applications including the existence of dynamics in the thermodynamic limit [8, 33], simulation of (real-time) evolution with local Hamiltonians [15], and mainly to study features of ground states of local Hamiltonians, namely multi-dimensional Lieb–Schultz–Mattis theorems [16], exponential clustering property [20, 34], area laws [18] or classification of phases [5], to name a few; see [19, 35–37] and references therein.

Lieb–Robinson type estimates for complex time evolution have been also investigated, see e.g. the works of Robinson [41] and Araki [1] both for finite-range interactions. The latter applies to one-dimensional spin systems and establishes that the infinite-volume time evolution operator applied to a local observable is analytic in the time variable on the whole complex plane, and the support of the evolved observable is approximately bounded but grows exponentially in the modulus of the complex time variable. These estimates become particularly useful when combined with Araki–Dyson expansionals [2] to deal with local perturbations of equilibrium states. Applications to one-dimensional spin systems include the absence of phase transition at every temperature for translationally invariant and finite range interactions [1], large deviations principles [27, 38, 39], central limit theorems [31, 32] and approximation of Gibbs states by MPOs [25]; see also [8, 44].

In this article, we aim to extend Araki’s result to a wider class of interactions.

In Sect. 2 we recall more recent versions of Robinson’s locality estimates for complex-time evolution [8, 41, 44] that apply to general lattices and interactions providing a disk around the origin where locality estimates hold. This turns out to be basically optimal for  with  as shown by Bouch [6], but contrasts with Araki’s result [1] in one-dimensional systems. To overcome this, we next provide a version in terms of the energy interaction across surfaces (Theorem 2.2), that applied to the case of bounded interactions in 1D leads to the main result of the paper (Theorem 2.3). In particular, for finite range interactions we recover Araki’s result, and for interactions that decay exponentially fast we get locality and analyticity on a disk around the origin whose radius scales with the exponent of the decay. We finish the section by showing that in combination with the usual Lieb–Robinson bounds, the previous locality estimates can be extended to a horizontal strip around the real axis of width equal to the diameter of the disk (Theorem 2.4 and Corollary 2.5).

To motivate these results, we illustrate two applications. The first one is to extend Araki’s result on equilibrium states by showing that, under translational invariance, the infinite volume Gibbs state has exponential decay of correlations for every temperature greater than the inverse of the width of the aforementioned strip. In Sect. 3 we introduce and present an auxiliary result on expansionals, and in Sect. 4 we detail the argument for the absence of phase transition along the lines of [1, 14, 30].

The second application, and actually main motivation of this work, deals with the spectral gap problem for parent Hamiltonians of Projected Entangled Pair States (PEPS). In the recent article [22] the authors have proved that for PEPS in 2D, if the boundary states on rectangles correspond to Gibbs states whose Hamiltonians feature *nice* locality properties, then the parent Hamiltonian of the PEPS is gapped. Their proof requires that the boundary Hamiltonians are finite range, leaving open the case of interactions with exponential decay, that seems to concur better with numerical simulations in certain models [9]; as well as faster-than-exponential decay, that has been observed in the PEPS representation of thermal states in quantum double models [29]. In Sect. 5 we extend the result from to [22] to the faster-than-exponential case, and partially to the exponential case (namely, if the exponent of the interaction decay is large enough).

### Notation and terminology

Let us recall the standard notation on quantum spin systems over a countable and locally finite metric space $({\mathcal {V}},{\text {dist}})$, although we will usually consider only the *g*-dimensional lattice  or the periodic lattice  for some  endowed with the graph distance. At each site $x \in \mathcal {V}$ let us consider a local finite-dimensional Hilbert space , so for each finite subset  we have the corresponding Hilbert space  and algebra of observables , that is, the space of bounded and linear operators on . The assignment $X mapsto \mathcal {A}_{X}$ is actually monotonic, in the sense that for two finite sets $X \subset Y \subset \mathcal {V}$ we can identify through a canonical linear isometryWith this identification we have a directed set $(\mathcal {A}_{X})_{X}$ whose direct limit is the so-called *algebra of local observables* $\mathcal {A}_{loc}$. In particular, since the above inclusions are isometries, $\mathcal {A}_{loc}$ is endowed with a natural norm becoming a normed $*$-algebra containing each $\mathcal {A}_{X}$ isometrically. The completion of $\mathcal {A}_{loc}$, denoted $\mathcal {A}_{\mathcal {V}}$ or simply $\mathcal {A}$, is the *C*$^*$-*algebra of observables*. For a (maybe infinite) subset $\Lambda \subset \mathcal {V}$ we denote by $\mathcal {A}_{\Lambda }$ the closed subspace generated by all $\mathcal {A}_{X}$ with $X \subset \Lambda$ finite, and say that $Q \in \mathcal {A}$ has *support* in $\Lambda$ if $Q \in \mathcal {A}_{\Lambda }$.

Let us denote the partial trace over a finite subset $\Lambda$ by ${\text {Tr}}_{\Lambda }: \mathcal {A}_{\mathcal {V}} \longrightarrow \mathcal {A}_{\mathcal {V} {\setminus } \Lambda }$ and its normalized version as ${\textrm{tr}}_{\Lambda }= {\text {Tr}}_{\Lambda }/d^{|\Lambda |}$. The tracial state over ${\mathcal {A}}$ will be simply denoted as ${\textrm{tr}}$.

Let $\Phi$ be a local interaction on the system, namely a map which associates to each (non-empty) finite subset $X \subset {\mathcal {V}}$ an element $\Phi _{X} = \Phi _{X}^{\dagger } \in \mathcal {A}_{X}$. We denote for each $n \ge 0$

$$\begin{aligned} \Omega _{n} := \sup _{x \in \mathcal {V}}{ \,\, \sum _{\begin{array}{c} X \ni x \,\,\, \text {s.t.}\\ {\text {diam}}(X) \ge n \end{array}}{ \Vert \Phi _{X}\Vert }}, \end{aligned}$$

where ${\text {diam}}(X)$ denotes the diameter of *X*. We will assume that $\Omega _{0}$ is finite, condition sometimes referred as $\Phi$ being a *bounded interaction*. The sequence $(\Omega _{n})_{n}$ is non-increasing and quantifies the decay of the interactions between sites. We say that $\Phi$ has *finite range* if there is $r>0$ such that $\Omega _{n}=0$ whenever $n>r$, or that $\Phi$ has *exponential decay* if there exists $\lambda > 0$ such that

$$\begin{aligned} \Vert \Phi \Vert _{\lambda }:= \sum _{n \ge 0}{\Omega _{n} \, e^{\lambda n}} \, < \,\infty . \end{aligned}$$

For each finite subset $\Lambda \subset \mathcal {V}$ we define the Hamiltonian

$$\begin{aligned} H_{\Lambda }:= \sum _{X \subset \Lambda }{\Phi _{X}}. \end{aligned}$$

The corresponding time-evolution operator in the complex variable $s \in \mathbb {C}$ is then given by

1 $$\begin{aligned} \Gamma _{H_\Lambda }^{s}(Q) = \Gamma ^{s}_{\Lambda }(Q) = e^{isH_{\Lambda }} Q e^{-isH_{\Lambda }}\quad \text {for each}\quad Q \in \mathcal {A}, \end{aligned}$$

or equivalently, through the Dyson series

2 $$\begin{aligned} \Gamma _{\Lambda }^{s}(Q) = \sum _{m=0}^{\infty }{\frac{s^{m}}{m!} \, \delta _{H_{\Lambda }}^{m}(Q)}, \end{aligned}$$

where $\delta _{H_{\Lambda }}(Q) := i [H_{\Lambda }, Q]$ is the commutator operator. Notice that this series converges absolutely whenever $\Lambda$ is finite. When considering the time evolution operator on the whole system (whenever it can be defined) we will simply write $\Gamma ^{s}$ or $\Gamma _{H}^{s}$.

For the one-dimensional lattice $\mathbb {Z}$, given $j \in \mathbb {Z}$ let us denote by $\tau _{j}$ the *lattice translation* homomorphism on $\mathcal {A}_{\mathbb {Z}}$ defined for each $Q \in M_{d}(\mathbb {C})$ by

$$\begin{aligned} \tau _{j} \, Q^{(k)} = Q^{(j+k)}, \end{aligned}$$

where $Q^{(k)} \in \mathcal {A}_{\{ k\}}$ is the element that coincides with *Q* on site *k* and with the identity on the rest. We say that the interaction $\Phi$ is *translationally invariant* if for every finite subset $X \subset \mathbb {Z}$ and every $j \in \mathbb {Z}$

$$\begin{aligned} \tau _{j} \, \Phi _{X} = \Phi _{j + X}. \end{aligned}$$

This nomenclature also applies to the one-sided version $\mathcal {A}_{\mathbb {N}} = \mathcal {A}_{[1,\infty )}$. In this setup, for each $Q \in \mathcal {A}_{\mathbb {N}}$, $m \in \mathbb {N}$ and real number $x>1$ define

$$\begin{aligned} \Vert Q\Vert _{n}&:= \inf \{ \Vert Q - Q_{n}\Vert :Q_{n} \in \mathcal {A}_{[1,n]} \},\\ {\left| \left| \left| Q \right| \right| \right| }_{m,x}&:= \Vert Q\Vert + \sum _{n \ge m}{\Vert Q\Vert _{n} \; x^{n}}. \end{aligned}$$

The vector subspace of $\mathcal {A}_{\mathbb {N}}$ given by

$$\begin{aligned} \mathcal {A}_{\mathbb {N}}(x) := \{ Q \in \mathcal {A}_{\mathbb {N}} :{\left| \left| \left| Q \right| \right| \right| }_{1,x} < \infty \}\, \end{aligned}$$

turns out to be a Banach space when endowed with any of the norms ${\left| \left| \left| \cdot \right| \right| \right| }_{m,x}$.

We will denote $\mathbb {N}_{0} := \mathbb {N} \cup \{ 0\}$. For each $n \in \mathbb {N}$ and $\alpha = (\alpha _{1}, \ldots , \alpha _{n}) \in \mathbb {N}_{0}^{n}$ let us write $|\alpha | := \alpha _{1} + \cdots + \alpha _{n}$.

## Analytic Lieb–Robinson Bounds

Let us consider a quantum spin system over $\mathbb {Z}^{g}$ and a local interaction $\Phi$ with exponential decay. Given a local observable *A* with support in a finite set $\Lambda _{0} \subset \mathbb {Z}^{g}$, we intend to compare the norm of the difference between $\Gamma _{\Lambda '}^{s}(A)$ and $\Gamma _{\Lambda }^{s}(A)$ on two larger sets $\Lambda ' \supset \Lambda \supset \Lambda _{0}$, in order to analyze the region and rate of convergence of the sequence of operators $s \mapsto \Gamma _{\Lambda _{n}}^{s}(A)$ where $\Lambda _{n}$ is an increasing and absorbing sequence of subsets of $\mathbb {Z}^{g}$.

For real values, it is well-known that the (global) real-time evolution operator $\Gamma ^{t}$ is well-defined for every $t \in \mathbb {R}$ and that the sequence of automorphisms $\mathbb {R} \ni t \mapsto \Gamma _{\Lambda _{n}}^{t}$ indexed on all finite subsets $\Lambda \subset \mathbb {Z}^{g}$ satisfies, for every quasi-local observable *A*, that3uniformly for *t* in compact intervals, see e.g. [8]. Indeed, Lieb–Robinson bounds [28] can be applied to show that there are constants $C,\mu ,v >0$, independent of *A*, such that

$$\begin{aligned} \Vert \Gamma _{\Lambda '}^{t}(A) - \Gamma _{\Lambda }^{t}(A) \Vert \, \le \, C \, \Vert A\Vert \, |\Lambda _{0}| \, e^{v|t|} \, e^{- \mu {\textrm{dist}}\,(\Lambda _{0}, \mathbb {Z}^{g} {\setminus } \Lambda )}, \end{aligned}$$

see [33, Theorem 2.2] and [8, Theorem 6.2.11]. These results implicitly use that the maps $\Gamma ^{t}_{\Lambda }$ are norm-preserving as *t* is real, so they cannot be applied to the complex-valued case.

However other arguments do not explicitly use this fact, leading to results also for complex values. This is the case of the proof of Robinson [41] for interactions such that $\Phi _{X} = 0$ whenever |*X*| is larger than a prefixed value. This was later extended to a setting in which interactions $\Phi _{X}$ decay exponentially fast with the cardinal or the diameter of *X* in works of Ruelle [44, Lemma 7.6.1 and Theorem 7.6.2] and Bratteli and Robinson [8, Theorem 6.2.4]. In the latter, it is shown that in the case of a general lattice $\mathbb {Z}^{g}$ and interactions $\Phi$ satisfying

$$\begin{aligned} \Vert \Phi \Vert _{\lambda }^{(1)}:= \sum _{n \ge 0} e^{\lambda n} \Big ( \, \sup _{x \in \mathbb {Z}^{g}} \sum _{\begin{array}{c} X \ni x \\ |X| = n+1 \end{array}} \Vert \Phi (X)\Vert \, \Big ) < \infty \end{aligned}$$

for some $\lambda >0$, then the (global) time evolution operator $s \mapsto \Gamma ^{s}(Q)$ is analytic and quasi-local (with exponential tails) on the disk

4 $$\begin{aligned} |z| < \frac{\lambda }{ 2 \, \Vert \Phi \Vert ^{(1)}_{\lambda }}. \end{aligned}$$

The advantage of this result is that it applies to lattices of arbitrary dimensions. Intuitively, one might expect that the size of the region of analyticity grows with $\lambda$, maybe approaching the whole complex plane as $\lambda$ tends to infinity. However, even if $\Vert \Phi \Vert _{\lambda }^{(1)} < \infty$ for every $\lambda >0$, the lower estimate

$$\begin{aligned} \Vert \Phi \Vert _{\lambda }^{(1)} \ge \Omega _{0} + (e^{\lambda }-1) \Omega _{1}\, \end{aligned}$$

yields that

$$\begin{aligned} |z|<\frac{\lambda }{ 2 \, \Vert \Phi \Vert ^{(1)}_{\lambda }} \le \frac{\lambda }{2 \Omega _{0} + 2(e^{\lambda } - 1) \Omega _{1}}. \end{aligned}$$

Thus, the radius is uniformly bounded on $\lambda$ unless $\Omega _{1}=0$, that is, unless there are no interactions between different sites.

In the case of $\mathbb {Z}^{g}$ with $g \ge 2$, this feature can be considered tight, since according to [6] we cannot expect convergence in the whole complex plane, not even for $g=2$ and translation invariant nearest neighbor interactions. However, in the case of $\mathbb {Z}$ we should expect a stronger result, since Araki [1] proved analyticity and quasi-locality on the whole complex plane in case of finite-range interactions.

### Theorem 2.1

(Informal). Let $\Phi$ be an interaction on a quantum spin system over $\mathbb {Z}$ such that $\Vert \Phi \Vert _{\lambda } < \infty$ for some $\lambda >0$. Then, for every local observable *A* the map $s \mapsto \Gamma ^{s}(A)$ is well-defined, analytic and quasi-local (with exponential tails) on the disk

5 $$\begin{aligned} |z|<\frac{\lambda }{4 \Omega _{0}} . \end{aligned}$$

Let us stress again a main difference with respect to other estimates such as (4): here the bound grows to infinity with $\lambda$, and allows to deduce analyticity on the whole complex plane if interactions decay faster than any exponential, recovering in particular Araki’s result for finite-range interactions. Combining this result with the ordinary Lieb–Robinson bounds one can moreover extend this result to the whole strip $|{\text {Im}}(s)|<\lambda /(4 \Omega _{0})$, see Corollary 2.5.

### Energy across surface

The next locality estimates involve properties of the energy interaction across surfaces, see [8, pp. 249–251]. Notice that the statement does not depend on the metric distance, so it may be applicable to a wider variety of situations.

#### Theorem 2.2

Let us consider a quantum spin system over $\mathcal {V}$ with bounded interaction $\Phi$, and fix an increasing sequence $(\Lambda _{n})_{n \ge 0}$ of finite subsets of $\mathcal {V}$. For each $0 \le j < k$ let us denote

$$\begin{aligned} W(j,k) := \sum _{\begin{array}{c} X \cap \Lambda _{j} \ne \emptyset \,\,\text {and} \\ X \cap (\Lambda _{k} {\setminus } \Lambda _{k-1}) \ne \emptyset \end{array}} \Vert \Phi _{X}\Vert , \quad W(j,j) := \sum _{X \cap \Lambda _{j} \ne \emptyset }{\Vert \Phi _{X} \Vert } . \end{aligned}$$

Then, for every local observable $A \in \mathcal {A}$ with support in $\Lambda _{0}$ and every $0 \le \ell \le L$

6 $$\begin{aligned} \Vert \Gamma _{\Lambda _{L}}^{s}(A)\Vert \,&\le \, \Vert A\Vert \, \sum _{k=0}^{L}{e^{2 |s| \Omega _{0} |\Lambda _{k}|} \, W^{*}_{k}(2|s|)} , \end{aligned}$$

7 $$\begin{aligned} \Vert \Gamma _{\Lambda _{L}}^{s}(A) - \Gamma _{\Lambda _{\ell }}^{s}(A)\Vert \,&\le \, \Vert A\Vert \, \sum _{k=\ell +1}^{L}{e^{2 |s| \Omega _{0} |\Lambda _{k}|} \, W^{*}_{k}(2|s|)} , \end{aligned}$$

where we are denoting for each $x \ge 0$

$$\begin{aligned} W^{*}_{0}(x) := 1, \quad W^{*}_{k}(x) := \sum _{n=1}^{\infty } \left( \sum _{0 = \beta _{0}< \cdots < \beta _{n}=k} \,\, \prod _{j=1}^{n} W(\beta _{j-1}, \beta _{j}) \right) \frac{x^{n}}{n!}\quad (k \ge 1). \end{aligned}$$

Remark that $W_{k}^{*}(x)$ is actually a finite sum over $1 \le n \le k$, since for $n>k$ the corresponding summand is zero (empty).

#### Proof

Let us assume that $\Vert A\Vert = 1$. We are going to prove first that (7) holds for consecutive regions $\Lambda _{k}$ and $\Lambda _{k-1}$. We make use of the estimate

8 $$\begin{aligned} \Vert \Gamma _{\Lambda _{k}}^{s}(A) - \Gamma _{\Lambda _{k-1}}^{s}(A) \Vert \le \sum _{m=1}^{\infty } \, \frac{|s|^{m}}{m!} \Vert \delta _{H_{\Lambda _{k}}}^{m}(A) - \delta _{H_{\Lambda _{k-1}}}^{m}(A) \Vert . \end{aligned}$$

We must then find good estimates of the summands in the right-hand side of (8). The argument is split into several stages.

**Step I**: *Let us denote for every* $m \in \mathbb {N}$

$$\begin{aligned} \mathcal {U}_{0}^{(m)}:= \delta _{H_{\Lambda _{0}}}^{m} (A), \quad \mathcal {U}_{k}^{(m)}:= \delta _{H_{\Lambda _{k}}}^{m} (A) - \delta _{H_{\Lambda _{k-1}}}^{m}(A) \quad (k > 0) . \end{aligned}$$

*Then, for every* $k \ge 0$

9 $$\begin{aligned} \mathcal {U}_{k}^{(m+1)} = \delta _{H_{\Lambda _{k}}} (\mathcal {U}_{k}^{(m)} )+ \sum _{i=0}^{k-1} \, \delta _{H_{\Lambda _{k}} - H_{\Lambda _{k-1}}} (\mathcal {U}_{i}^{(m)}), \end{aligned}$$

*where for* $k=0$ *the finite series on the right-hand side of* (9) *is equal to zero*. To prove this statement, note that fixed $m \ge 1$ and $k \ge 0$ we can decompose

$$\begin{aligned} \delta _{H_{\Lambda _{k}}}^{m}(A) = \sum _{i=0}^{k}{\mathcal {U}_{i}^{(m)}}. \end{aligned}$$

Applying the operator $\delta _{H_{\Lambda _{k}}}$ on both sides and using that $\delta _{H_{\Lambda _{j}} - H_{\Lambda _{j-1}}} = \delta _{H_{\Lambda _{j}}} - \delta _{H_{\Lambda _{j-1}}}$, we get thatUsing this identity, we can immediately check that

$$\begin{aligned} \delta _{H_{\Lambda _{k}}}^{m+1} (A) - \delta _{H_{\Lambda _{k-1}}}^{m+1}(A)\, = \, \delta _{H_{\Lambda _{k}}} \, (\mathcal {U}_{k}^{(m)}) + \sum _{i=0}^{k-1}{\delta _{H_{\Lambda _{k}} - H_{\Lambda _{k-1}}} \, (\mathcal {U}_{i}^{(m)}}), \end{aligned}$$

which finishes the proof of the statement.

**Step II**: *Define for every* $m \ge 1$ *and* $k \ge 0$ *the non-negative number*

10 $$\begin{aligned} \phi (m,k):= \sum _{ 0 = \alpha _{0} \le \cdots \le \alpha _{m} = k}{\prod _{j=1}^{m}{ W(\alpha _{j-1}, \alpha _{j}) }}. \end{aligned}$$

*Then, we have*

11 $$\begin{aligned} \Vert \mathcal {U}_{k}^{(m)} \Vert \, \le \, 2^{m} \, \phi (m,k). \end{aligned}$$

To prove this statement, let us first note that if $0 \le j < k$ and *B* is an observable with support in $\Lambda _{j}$, then

12 $$\begin{aligned} \Vert \delta _{H_{\Lambda _{j}}} (B)\Vert \, \le \, \sum _{X \cap \Lambda _{j} \ne \emptyset }{ \Vert \delta _{\Phi _{X}} (B) \Vert } \, \le \, 2 \, \Vert B\Vert \, W(j,j) \end{aligned}$$

and

13 $$\begin{aligned} \begin{aligned} \Vert \delta _{H_{\Lambda _{k}} - H_{\Lambda _{k-1}}} (B) \Vert \,&\le \, \sum _{\begin{array}{c} X \subset \Lambda _{k} \,\, \text {s.t.} \\ X \nsubseteq \Lambda _{k-1} \,\, \text {and}\,\, X \cap \Lambda _{j} \ne \emptyset \end{array}} { \Vert \delta _{\Phi _{X}}(B) \Vert } \, \le \, 2 \, \Vert B\Vert \, W(j,k) . \end{aligned} \end{aligned}$$

We are going to use these inequalities to show by induction on $m \ge 1$ that (11) holds for every $k \ge 0$. The case $m=1$ follows taking $j = 0$ and $B = A$ in (12) and (13). Fixed $m \ge 1$ and assuming that formula (11) holds for this *m* and every $k \ge 0$, we can apply the recursive formula (9) from Step I to get that for every $k \ge 0$

$$\begin{aligned} \Vert \mathcal {U}_{k}^{(m+1)} \Vert \,&\le \, \Vert \delta _{H_{\Lambda _{k}}} (\mathcal {U}_{k}^{(m)}) \Vert + \sum _{i=0}^{k-1} \Vert \delta _{H_{\Lambda _{k}} - H_{\Lambda _{k-1}}} (\mathcal {U}_{i}^{(m)})\Vert \\&\le \, 2 \, \Vert \mathcal {U}_{k}^{(m)}\Vert \, W(k,k) \, + \, \sum _{i=0}^{k-1} 2 \, \Vert \mathcal {U}_{i}^{(m)}\Vert \, W(i,k) \\&\le \, 2^{m+1} \, \sum _{i=0}^{k} \phi (m,i) \, W(i, k)\\&= \, 2^{m+1} \, \phi (m+1, k) . \end{aligned}$$

This finishes the proof of the statement.

**Step III**: We are going to upper bound $\phi (m,k)$ by a more manageable expression using that for every $j \ge 0$

$$\begin{aligned} W(j,j) \le \sum _{x \in \Lambda _{j}} \sum _{X \ni x} \Vert \Phi _{X} \Vert \le |\Lambda _{j}| \, \Omega _{0}. \end{aligned}$$

Indeed, for $k=0$ we immediately get that

14 $$\begin{aligned} \phi (m,0) = (W(0,0))^{m} \le (\Omega _{0} \, |\Lambda _{0}|)^{m}. \end{aligned}$$

In the case $k \ge 1$, we first rearrange the sum by gathering those terms corresponding to sequences $0 = \alpha _{0} \le \cdots \le \alpha _{m} = k$ that have the same range, namely

$$\begin{aligned} \phi (m,k) = \sum _{n=1}^{m} \,\, \sum _{0 = \beta _{0}< \cdots < \beta _{n} = k} \,\, \sum _{\begin{array}{c} 0 = \alpha _{0} \le \cdots \le \alpha _{m} = k \\ \{ \alpha _{0}, \ldots , \alpha _{m}\} = \{ \beta _{0}, \ldots , \beta _{n}\} \end{array}} \,\, \prod _{j=1}^{m}{W(\alpha _{j-1}, \alpha _{j})} . \end{aligned}$$

Then, we upper bound each factor $W(\alpha _{j-1}, \alpha _{j})$ with $\alpha _{j-1}=\alpha _{j}$ by $\Omega _{0} |\Lambda _{k}|$ using the aforementioned inequality. Note that fixed $0 = \beta _{0}< \cdots < \beta _{n} = k$, the number of sequences $0 = \alpha _{0} \le \cdots \le \alpha _{m} = k$ such that $\{ \alpha _{0}, \ldots , \alpha _{m}\} = \{ \beta _{0}, \ldots , \beta _{n} \}$ coincides with the number of subsets with cardinality *n* of $\{ 1, \ldots , m\}$. Thus,

15 $$\begin{aligned} \phi (m,k) \le \sum _{n=1}^{m}{\left( \sum _{0 = \beta _{0}< \cdots < \beta _{n} = k} \,\, \prod _{j=1}^{n}{W(\beta _{j-1}, \beta _{j})} \right) \Omega _{0}^{m-n} \, |\Lambda _{k}|^{m-n} \, \left( {\begin{array}{c}m\\ n\end{array}}\right) }. \end{aligned}$$

**Step IV**: Applying estimates (11) and (15) to (8), we conclude

$$\begin{aligned}&\Vert \Gamma _{\Lambda _{k}}^{s}(A) - \Gamma _{\Lambda _{k-1}}^{s}(A) \Vert \\&\quad \le \sum _{m=1}^{\infty } \frac{(2 |s|)^{m}}{m!} \, \sum _{n=1}^{m}{\left( \sum _{0 = \beta _{0}< \cdots< \beta _{n} = k} \,\, \prod _{j=1}^{n}{W(\beta _{j-1}, \beta _{j})} \right) \Omega _{0}^{m-n} \, |\Lambda _{k}|^{m-n} \, \left( {\begin{array}{c}m\\ n\end{array}}\right) }\\&\quad = \sum _{m=1}^{\infty } \sum _{n=1}^{m} \left( \sum _{0 = \beta _{0}< \cdots< \beta _{n} = k} \,\, \prod _{j=1}^{n}{W(\beta _{j-1}, \beta _{j})} \right) \, \frac{\Omega _{0}^{m-n} \, |\Lambda _{k}|^{m-n} (2 |s|)^{m-n}}{(m-n)!} \, \frac{(2 |s|)^{n}}{n!}\\&\quad \le \sum _{n=1}^{\infty } \frac{(2 |s|)^{n}}{n!} \, \left( \sum _{0 = \beta _{0}< \cdots< \beta _{n} = k} \,\, \prod _{j=1}^{n}{W(\beta _{j-1}, \beta _{j})} \right) \, \sum _{m=n}^{\infty } \frac{\Omega _{0}^{m-n} \, |\Lambda _{k}|^{m-n} (2 |s|)^{m-n}}{(m-n)!}\\&\quad = e^{2 |s| \Omega _{0} |\Lambda _{k}|} \, \sum _{n=1}^{\infty } \frac{(2 |s|)^{n}}{n!} \, \left( \sum _{0 = \beta _{0}< \cdots < \beta _{n} = k} \,\, \prod _{j=1}^{n}{W(\beta _{j-1}, \beta _{j})} \right) \\&\quad =e^{2|s|\Omega _{0}|\Lambda _{k}|} \, W_{k}^{*}(2|s|). \end{aligned}$$

As a consequence, for every $0 \le \ell \le L$ we can use a telescopic sum to estimate

$$\begin{aligned} \Vert \Gamma _{\Lambda _{L}}^{s}(A) - \Gamma _{\Lambda _{\ell }}^{s}(A) \Vert \le \sum _{k=\ell +1}^{L} \Vert \Gamma _{\Lambda _{k}}^{s}(A) - \Gamma _{\Lambda _{k-1}}^{s}(A) \Vert \le \sum _{k=\ell +1}^{L} e^{2|s|\Omega _{0}|\Lambda _{k}|} \, W_{k}^{*}(2|s|). \end{aligned}$$

To prove (6), observe that by (14) and (11) we have

$$\begin{aligned} \Vert \Gamma ^{s}_{\Lambda _{0}}(A)\Vert \le \sum _{m=0}^{\infty } \frac{|s|^{m}}{m!} \Vert \mathcal {U}_{0}^{(m)}\Vert \, \le \, \sum _{m=0}^{\infty } \frac{(2|s|)^{m}}{m!}(\Omega _{0} |\Lambda _{0}| )^{m} = e^{2|s| \Omega _{0}|\Lambda _{0}|}\, W_{0}^{*}(2|s|). \end{aligned}$$

Hence, for every $L \ge 0$ we can use a telescopic sum to get

$$\begin{aligned} \Vert \Gamma ^{s}_{\Lambda _L}(A)\Vert \le \Vert \Gamma ^{s}_{\Lambda _0}(A)\Vert + \sum _{k=1}^{L} \Vert \Gamma _{\Lambda _{k}}^{s}(A) - \Gamma _{\Lambda _{k-1}}^{s}(A) \Vert \le \sum _{k=0}^{L} e^{2|s|\Omega _{0}|\Lambda _{k}|} \, W_{k}^{*}(2|s|). \end{aligned}$$

This finishes the proof of the theorem. $\square$

The previous result is not suitable for $\mathbb {Z}^{g}$ with $g\ge 2$. Indeed, consider a quantum spin system over $\mathbb {Z}^{2}$, an observable *A* supported at the origin, the sequence $\Lambda _{n} = [-n,n]^{2}$ and some nearest neighbour interaction with $W(j, j + 1)> \varepsilon > 0$ for every $j \ge 0$ and some $\varepsilon > 0$. Then, $W_{k}^{*}(2|s|) \ge (2\varepsilon |s|)^{k}/k!$ and $e^{2 |s| \Omega _{0} |\Lambda _{k}|} = e^{2|s|\Omega _{0} (2k+1)^{2}}$, so the series in (7) cannot converge as *L* tends to infinity unless *s* is equal to zero.

### One-dimensional lattice

We are going to particularize Theorem 2.2 to the one-dimensional lattice $\mathbb {Z}$. The natural supporting sets to consider here are intervals $J=[a,b]$. We denote $J_{k}:=[a-k,b+k]$ for every $k \in \mathbb {N}$, so that $|J_{k} {\setminus } J_{k-1}| \le 2$. Following the notation of the aforementioned theorem,

$$\begin{aligned} W(j,k) \le 2 \, \Omega _{k-j} \quad \text{ for } \text{ every } 0 \le j < k. \end{aligned}$$

This immediately yields the following main result.

#### Theorem 2.3

Let us consider a quantum spin system over $\mathbb {Z}$ with bounded interaction $\Phi$ and let $A \in \mathcal {A}$ be a local observable with support in an interval *J*. Then, for every $0 \le \ell \le L$ and every $s \in \mathbb {C}$

16 $$\begin{aligned} \Vert \Gamma ^{s}_{J_{L}}(A) - \Gamma ^{s}_{J_{\ell }}(A) \Vert&\,\, \le \,\, \Vert A\Vert \, e^{2 |s| \Omega _{0} |J|} \, \sum _{k=\ell + 1}^{L} e^{4 |s| \Omega _{0} k}\, \Omega _{k}^{*}(4 |s|), \end{aligned}$$

17 $$\begin{aligned} \Vert \Gamma ^{s}_{J_{L}}(A) \Vert&\,\, \le \,\, \Vert A\Vert \, e^{2 |s| \Omega _{0} |J|} \,\,\, \sum _{k=0}^{L} e^{4 |s| \Omega _{0} k}\, \Omega _{k}^{*}(4 |s|), \end{aligned}$$

where we are denoting for each $x \ge 0$

18 $$\begin{aligned} \Omega _{0}^{*}(x) = 1, \quad \Omega _{k}^{*}(x) = \sum _{n=1}^{k}{ \Big ( \sum _{\begin{array}{c} \alpha \in \mathbb {N}^{n}\\ |\alpha | = k \end{array}} \, \prod _{j=1}^{n}\Omega _{\alpha _{j}} \Big ) \frac{x^{n}}{n!} } . \end{aligned}$$

Notice that the $\Omega _{k}^{*}(x)$’s are actually the coefficients in the power series expansion of

19 $$\begin{aligned} \mathbb {D} \ni z \, \longmapsto \, \exp {\left[ x \, \sum _{k=1}^{\infty } \Omega _{k} z^{k}\right] } \, = \, \sum _{k = 0}^{\infty }{\Omega _{k}^{*}(x) \, z^{k}} . \end{aligned}$$

In the rest of the section, we shed more light on the bounds in the previous theorem by particularizing them to interactions with finite range and exponential decay. Next, we reformulate the usual Lieb–Robinson bounds for real time evolution in terms of the sequence $\Omega _{n}$ to compare it with the previous result, and finally present a combined locality estimate for real and imaginary time evolution.

#### Finite-range interactions

Assume that there exists $r \ge 2$ such that $\Omega _{k} = 0$ if $k \ge r$ and let us estimate $\Omega _{k}^{*}(x)$. Note that if $\alpha \in \mathbb {N}^{n}$ satisfies $|\alpha | = k \ge r n$ then $\alpha _{j} \ge r$ for some *j*, and so $\Omega _{\alpha _{1}} \cdot \ldots \cdot \Omega _{\alpha _{n}} =0$. Hence we can restrict the range of *n* in formula (18) to

$$\begin{aligned} \Omega _{k}^{*}(x) = \sum _{k/r< n \le k} \Big ( \sum _{\begin{array}{c} \alpha \in \mathbb {N}^{n}\\ |\alpha | = k \end{array}} \, \prod _{j=1}^{n}\Omega _{\alpha _{j}} \Big ) \frac{x^{n}}{n!} \le \sum _{k/r <n \le k} \frac{(x\Omega _{0}re)^{n}}{n!} \end{aligned}$$

where in the last inequality we have used that $\Omega _{k} \le \Omega _{0}$ and that the number of elements $\alpha \in \mathbb {N}^{n}$ with $|\alpha |=k$ is $$\left( {\begin{array}{c}k-1\\ n-1\end{array}}\right) = \frac{n}{k} \left( {\begin{array}{c}k\\ n\end{array}}\right) \le \left( \frac{k e}{n}\right) ^{n} \le (re)^{n}$$ whenever $k<rn$. Thus, we can estimate in (16)

$$\begin{aligned} \sum _{k= \ell + 1}^{L}{e^{x \Omega _{0} k} \, \Omega ^{*}_{k}(x)}&\le \sum _{k=\ell + 1}^{L} \, \sum _{k/r< \, n \, \le k} \, e^{x \Omega _{0} k} \, \frac{(x\Omega _{0}re)^{n}}{n!} \, \le \, \sum _{n>\ell /r} \,\, \sum _{n \le k <rn} e^{x \Omega _{0}k} \frac{(x\Omega _{0}re)^{n}}{n!}\\&\le \, \sum _{n>\ell /r} r^{n} e^{x \Omega _{0}nr} \frac{(x\Omega _{0}re)^{n}}{n!} \, = \, \sum _{n > \ell /r} \left( x \Omega _{0} r^{2} e^{1+x \Omega _{0} r} \right) ^{n}\frac{1}{n!}\\&\le \exp {\left( x \Omega _{0} r^{2} e^{1+x\Omega _{0} r} \right) } \, \frac{\left( x \Omega _{0} r^{2} e^{1+x\Omega _{0} r} \right) ^{[\ell /r] + 1}}{\left( [\ell /r]+1 \right) !}. \end{aligned}$$

This estimate shows that for every observable *A* with support in a compact interval *J*, the sequence of maps $s \mapsto \Gamma _{J_{\ell }}^{s}(A)$ converges superexponentially fast as $\ell$ tends to infinity on every bounded subset of the complex plane. Indeed, the asymptotic order of convergence coincides with the one provided by Araki in [1, Theorem 4.2].

#### Exponentially decaying interactions

Let us assume that our interaction has exponential decay $\Vert \Phi \Vert _{\lambda } < \infty$ for some $\lambda > 0$. Then, using (19) we deduce that every $0 \le x \le \frac{\lambda }{\Omega _{0}}$

20 $$\begin{aligned} \begin{aligned} \sum _{k=\ell + 1}^{L}{e^{x \Omega _{0} k} \, \Omega _{k}^{*}(x)}&\le e^{(x \Omega _{0} - \lambda ) \ell } \sum _{k=\ell +1}^{L} e^{\lambda k} \Omega _{k}^{*}(x) \, \le e^{(x \Omega _{0} - \lambda )\ell } \, e^{ x \Vert \Phi \Vert _{\lambda } } . \end{aligned} \end{aligned}$$

Combining this estimate with Theorem 2.3 we conclude that for every local observable *A* with support in an interval *J* and every $s \in \mathbb {C}$ with $|s| \le \frac{\lambda }{4 \Omega _{0}}$

21 $$\begin{aligned} \Vert \Gamma ^{s}_{J_{L}}(A) - \Gamma ^{s}_{J_{\ell }}(A) \Vert&\,\, \le \,\, \Vert A\Vert \, e^{2|s|\Omega _{0}|J|} \,\, e^{ 4|s| \, \Vert \Phi \Vert _{\lambda } }\,\, e^{(4|s|\Omega _{0} - \lambda ) \ell }, \end{aligned}$$

22 $$\begin{aligned} \Vert \Gamma ^{s}_{J_{L}}(A) \Vert&\,\, \le \,\, \Vert A\Vert \, e^{2|s|\Omega _{0}|J|} \,\, e^{ 4|s| \, \Vert \Phi \Vert _{\lambda } }. \end{aligned}$$

As a consequence, the sequence $s \mapsto \Gamma ^{s}_{J_{\ell }}(A)$ converges as $\ell$ tends to infinite on every compact subset of the open disk centered at the origin and radius $\lambda /(4 \Omega _{0})$ exponentially fast, leading to the statement of Theorem 2.1.

#### Lieb–Robinson bounds

Let us illustrate how the Lieb–Robinson bounds for real time evolution can be reformulated in terms of the sequence $\Omega _{k}$. They are significantly better than the complex time version from Theorem 2.3.

##### Theorem 2.4

Let us consider a quantum spin system over $\mathbb {Z}$ with local bounded interaction $\Phi$ and let *A* be a local observable with support in a finite set $\Lambda _{0} \subset \mathbb {Z}$. Then, for every $0 \le \ell \le L$ and every $t \in \mathbb {R}$ we have

$$\begin{aligned} \Vert \Gamma _{\Lambda _{L}}^{t}(A) - \Gamma _{\Lambda _{\ell }}^{t}(A) \Vert \, \le \, \Vert A\Vert \, |\Lambda _{0}|\, e^{4 |t| \Omega _{0}} \, \sum _{k \ge \ell + 1} \, \Omega _{k}^{*}(4 |t|). \end{aligned}$$

where we are denoting $\Lambda _{\ell } := \{ x \in \mathbb {Z} :{\text {dist}}(x, \Lambda _{0}) \le \ell \}$.

##### Proof

Let us recall that by [33, Section 2.1], for every finite subset $\Lambda \subset \mathbb {Z}$, every local observable *B* with support in a set $\mathfrak {B} \subset \mathbb {Z}$ and every $t \in \mathbb {R}$

$$\begin{aligned} \Vert [\Gamma ^{t}_{\Lambda }(A), B] \Vert \le \Vert A\Vert \, \Vert B\Vert \, \sum _{k=0}^{\infty }{\frac{(2|t|)^{k}}{k!} \, a_{k}}, \end{aligned}$$

where $a_{0} = 1$ and for each $k \ge 1$

$$\begin{aligned} a_{k} = \sum _{\begin{array}{c} X_{1} \cap \Lambda _{0} \ne \emptyset \end{array}} \,\, \sum _{ X_{2} \cap X_{1} \ne \emptyset } \,\, \ldots \, \sum _{ \begin{array}{c} X_{k} \cap X_{k-1} \ne \emptyset \\ X_{k} \cap \mathfrak {B} \ne \emptyset \end{array}} \,\, \prod _{j=1}^{k}\Vert \Phi _{X_{j}}\Vert , \end{aligned}$$

where the sum is extended over subsets $X_{j} \subset \Lambda$. Combining this with the following inequality (see e.g. [33, Section 2.2])

$$\begin{aligned} \Vert \Gamma _{\Lambda _{L}}^{t}(A) - \Gamma _{\Lambda _{\ell }}^{t}(A) \Vert \, \le \, \sum _{\begin{array}{c} X \cap \Lambda _{\ell }^{c} \ne \emptyset \\ X \subset \Lambda _{L} \end{array}} \,\, \int _{0}^{|t|}{\Vert [ \Gamma _{\Lambda _{\ell }}^{u}(A), \Phi _{X} ] \Vert }\, d u, \end{aligned}$$

we can estimate

23 $$\begin{aligned} \Vert \Gamma _{\Lambda _{L}}^{t}(A) - \Gamma _{\Lambda _{\ell }}^{t}(A) \Vert \, \le \, \Vert A \Vert \, \sum _{k=1}^{\infty }{\frac{(2 |t|)^{k}}{k!} b_{k}}, \end{aligned}$$

where

$$\begin{aligned} b_{k} \,&= \sum _{X_{1} \cap \Lambda _{0} \ne \emptyset } \,\, \sum _{ X_{2} \cap X_{1} \ne \emptyset } \,\, \ldots \, \sum _{X_{k-1} \cap X_{k-2} \ne \emptyset } \,\, \sum _{\begin{array}{c} X_{k} \cap X_{k-1} \ne \emptyset \\ X_{k} \cap \Lambda _{\ell }^{c} \ne \emptyset \end{array}} \,\, \Vert \Phi _{X_{1}}\Vert \, \ldots \, \Vert \Phi _{X_{k}}\Vert \\&\le \, \sum _{j_{0} \in \Lambda _{0}} \,\, \sum _{j_{1}, \ldots , j_{k-1} \in \mathbb {Z}} \,\, \sum _{j_{k} \in \Lambda _{\ell }^{c}} \,\, \sum _{X_{1} \ni j_{0}, j_{1}} \, \ldots \sum _{X_{k} \ni j_{k-1}, j_{k}} \,\, \Vert \Phi _{X_{1}}\Vert \, \ldots \, \Vert \Phi _{X_{k}}\Vert \\&\le \, \sum _{j_{0} \in \Lambda _{0}} \,\, \sum _{j_{1}, \ldots , j_{k-1} \in \mathbb {Z}} \,\, \sum _{j_{k} \in \Lambda _{\ell }^{c}} \,\, \Omega _{|j_{1} - j_{0}|} \cdot \ldots \cdot \Omega _{|j_{k} - j_{k-1}|}\\&\le 2^{k} \, |\Lambda _{0}| \, \sum _{\alpha \in \mathbb {N}_{0}^{k}, \, |\alpha |> \ell } \Omega _{\alpha _{1}} \cdot \ldots \cdot \Omega _{\alpha _{k}}\\&\le 2^{k} \, |\Lambda _{0}| \, \sum _{j=1}^{k} \, \Big ( \sum _{\alpha \in \mathbb {N}^{j}, \, |\alpha | > \ell } \Omega _{\alpha _{1}} \cdot \ldots \cdot \Omega _{\alpha _{j}} \Big ) \, \Omega _{0}^{k-j} \, \left( {\begin{array}{c}k\\ j\end{array}}\right) . \end{aligned}$$

Arguing now as in Step IV of the proof of Theorem 2.2, we conclude the result $\square$

Finally, we present a locality estimate which combines the complex time version with the Lieb–Robinson bounds.

##### Corollary 2.5

Let us consider a quantum spin system over $\mathbb {Z}$ with interaction $\Phi$ having exponential decay $\Vert \Phi \Vert _{\lambda } < \infty$ for some $\lambda >0$. Then, for every local observable *A* with support in an interval *J*, every $1 \le \ell \le L$ and every complex number $s = t + i \beta \in \mathbb {C}$ we have

$$\begin{aligned} \Vert \Gamma _{J_{L}}^{s}(A) - \Gamma _{J_{\ell }}^{s}(A) \Vert \, \le \, 2 \, |J| \, e^{8|s| \, \Vert \Phi \Vert _{\lambda }} \, e^{2|\beta | \Omega _{0} |J|} \, \ell \, e^{(4|\beta | \Omega _{0} - \lambda ) \, \lfloor \ell /2 \rfloor } . \end{aligned}$$

In particular, the time evolution operator $s \longmapsto \Gamma ^{s}(A)$ is well-defined and quasilocal on the strip $\{ s \in \mathbb {C} :|{\text {Im}}(s)| < \lambda /(4 \Omega _{0})\}$.

##### Proof

Let us assume that $\Vert A\Vert = 1$ and fix $j:= \lfloor \ell / 2 \rfloor$. Then,

$$\begin{aligned}&\Vert \Gamma _{J_{L}}^{s} (A) - \Gamma _{J_{\ell }}^{s}(A) \Vert \\&\quad = \Vert \Gamma _{J_{L}}^{t} \Gamma _{J_{L}}^{i \beta }(A) - \Gamma _{J_{L}}^{t} \Gamma _{J_{j}}^{i \beta }(A) + \Gamma _{J_{L}}^{t} \Gamma _{J_{j}}^{i \beta }(A) - \Gamma _{J_{\ell }}^{t} \Gamma _{J_{j}}^{i \beta }(A) + \Gamma _{J_{\ell }}^{t} \Gamma _{J_{j}}^{i \beta }(A) - \Gamma _{J_{\ell }}^{t}\Gamma _{J_{\ell }}^{i \beta }(A) \Vert \\&\quad \le \, \Vert \Gamma _{J_{L}}^{i \beta }(A) - \Gamma _{J_{j}}^{i \beta }(A)\Vert + \Vert (\Gamma ^{t}_{J_{L}} - \Gamma ^{t}_{J_{\ell }}) \Gamma _{J_{j}}^{i \beta }(A) \Vert + \Vert \Gamma _{J_{\ell }}^{i \beta }(A) - \Gamma _{J_{j}}^{i \beta }(A)\Vert . \end{aligned}$$

The first and third summands can be bounded using (21) as

$$\begin{aligned} \Vert \Gamma _{J_{L}}^{i \beta }(A) - \Gamma _{J_{j}}^{i \beta }(A)\Vert + \Vert \Gamma _{J_{\ell }}^{i \beta }(A) - \Gamma _{J_{j}}^{i \beta }(A)\Vert \, \le \, 2 \, e^{2|\beta | \Omega _{0} |J|} \, e^{4|\beta | \, \Vert \Phi \Vert _{\lambda }} \, e^{(4|\beta |\Omega _{0} - \lambda )j}. \end{aligned}$$

The second summand can be estimated using Theorem 2.4 and (22),

$$\begin{aligned} \Vert (\Gamma ^{t}_{J_{L}} - \Gamma ^{t}_{J_{\ell }}) \Gamma _{J_{j}}^{i \beta }(A) \Vert \,&\, \le \, \Vert \Gamma _{J_{j}}^{i \beta }(A) \Vert \, |J_{j}| e^{4 |t| \Omega _{0}} \, e^{-\lambda (\ell - j)} e^{4|t| \, \Vert \Phi \Vert _{\lambda }}\\&\, \le \, e^{2|\beta | \Omega _{0} |J|} \, e^{4 |\beta | \, \Vert \Phi \Vert _{\lambda }} \, |J_{j}| \, e^{4 |t| \Omega _{0}} \, e^{-\lambda (\ell - j)} e^{4|t| \, \Vert \Phi \Vert _{\lambda }}\\&\, \le \, e^{6|s| \Omega _{0} |J|} \, e^{8|s| \, \Vert \Phi \Vert _{\lambda }} \, (\ell \, |J|) \, e^{- \lambda \, j}, \end{aligned}$$

where in the last inequality we have used that $\ell -j \ge j$ by definition. Combining these estimates we conclude the result. $\square$

## Local Perturbations and Expansionals

In perturbation theory we study for a given Hamiltonian *H* the effect of introducing a weak physical perturbance in the form of a new Hamiltonian *U*, namely $H + U$. It is then useful to relate $e^{- H}$ and $e^{-(H + U)}$ through identities of the form

$$\begin{aligned} e^{ - (H + U)} \, = \, E \, e^{ - H} \, E^{\dagger } \end{aligned}$$

for some suitable observable *E* featuring the locality properties *U*. Following Araki’s approach (see [1, p. 135] or [2]) we take $E = E_{r}(U/2, H/2)$ where24and its inverse25These identities are consequence of Duhamel’s formula and allow us to apply the locality results on imaginary time evolution from the previous sections to obtain the following result.

### Theorem 3.1

Let us consider a quantum spin system on $\mathbb {Z}$ with bounded interaction $\Phi$. For every $\beta > 0$, $a \in \mathbb {Z}$ and $p \ge 0$ let us define

$$\begin{aligned} E_{a,p}^{\beta }:= e^{-\beta H_{[a-p,a+1+p]}} \, e^{\beta H_{[a-p,a]} + \beta H_{[a+1,a+1+p]} }. \end{aligned}$$

Then, for every $0 \le p \le q$

$$\begin{aligned} \Vert E_{a,p}^{\beta }\Vert \,&\le \, \exp {\left( \, \frac{1}{2}\, \sum _{k =1}^{p+1} k \, e^{4 \Omega _{0} \beta k} \, \Omega _{k}^{*}(4 \beta ) \, \right) },\\ \Vert E_{a,p}^{\beta } - E_{a,q}^{\beta }\Vert&\le \, \frac{1}{2} \, \left( \sum _{k = p +2}^{q+1} k \, e^{4 \Omega _{0} \beta k} \, \Omega _{k}^{*}(4 \beta ) \, \right) \, \exp {\left( \frac{1}{2} \, \sum _{k =1}^{q+1} k \, e^{4 \Omega _{0} \beta k} \, \Omega _{k}^{*}(4 \beta ) \, \right) }. \end{aligned}$$

Moreover, the same inequalities hold if we replace $\Vert E_{a,p}^{\beta }\Vert$ and $\Vert E_{a,p}^{\beta } - E_{a,q}^{\beta }\Vert$ with $\Vert (E_{a,p}^{\beta })^{ -1}\Vert$ and $\Vert (E_{a,p}^{\beta })^{ -1} - (E_{a,q}^{\beta })^{ -1}\Vert$ respectively.

We can assume that $\beta = 1$ by absorbing $\beta$ in the interaction, since if we define $\Phi _{X}' = \beta \Phi _{X}$ for every finite $X \subset \mathbb {Z}$ the interaction $\Phi '$ satisfies for every $k \ge 0$

$$\begin{aligned} \Omega '_{k} = \beta \, \Omega _{k} \quad \text{ and } \quad \Omega '^{*}_{k}(x) = \Omega ^*_{k}(\beta x ). \end{aligned}$$

Denote $J:=[a,a+1]$. For each $p \ge 0$, let $J_{p}:=[a-p,a+1+p]$ and

$$\begin{aligned} U_{J_{p}} := \sum _{\begin{array}{c} X \subset J_{p} \,\, \text {s.t.} \,\, X \cap [a-p,a] \ne \emptyset \\ \,\,\, \text {and} \,\,X \cap [a+1,a+1+p] \ne \emptyset \end{array}} \Phi _{X}. \end{aligned}$$

With the notation of (24) and (25),

26 $$\begin{aligned} E_{a,p}^{1} = E_{l}(U_{J_{p}};H_{J_{p}}) \quad , \quad (E_{a,p}^{1})^{-1} =E_{r}(U_{J_{p}};H_{J_{p}}). \end{aligned}$$

The strategy of the proof is then clear.

### Lemma 3.2

With the previous notation, for every $0 \le p \le q$

27 $$\begin{aligned} \sup _{|s| \le 1} \Vert \Gamma _{H_{J_{p}}}^{s}(U_{J_{p}})\Vert&\, \le \, \frac{1}{2} \, \sum _{k = 1}^{p+1} k \, e^{4 \Omega _{0} k} \, \Omega _{k}^{*}(4), \end{aligned}$$

28 $$\begin{aligned} \sup _{|s| \le 1} \, \Vert \Gamma _{H_{J_{p}}}^{s}(U_{J_{p}}) - \Gamma _{H_{J_{q}}}^{s}(U_{J_{q}})\Vert&\, \le \, \frac{1}{2} \, \sum _{k = p +2}^{q+1} k \, e^{4 \Omega _{0} k} \, \Omega _{k}^{*}(4). \end{aligned}$$

### Proof

Let us start with the following observation: for each $m \ge 1$

$$\begin{aligned} \sum _{\begin{array}{c} j \ge 1, k \ge 0 \\ j+k=m \end{array}} \Omega _{k}^{*}(4) \Omega _{j}&= \Omega _{m}+ \sum _{\begin{array}{c} j \ge 1, k \ge 1 \\ j+k=m \end{array}} \sum _{n=1}^{k} \big ( \sum _{\begin{array}{c} \alpha \in \mathbb {N}^{n}\\ |\alpha |=k \end{array}} \Omega _{\alpha _{1}} \ldots \Omega _{\alpha _{n}} \Omega _{j}\big ) \frac{4^{n}}{n!} \\&\le \Omega _{m}+ \sum _{n=1}^{m-1} \big ( \sum _{\begin{array}{c} \alpha \in \mathbb {N}^{n+1}\\ |\alpha | = m \end{array}} \Omega _{\alpha _{1}} \ldots \Omega _{\alpha _{n+1}}\big ) \frac{4^{n}}{n!} \, \le \, \frac{m}{4} \Omega _{m}^{*}(4), \end{aligned}$$

so that for each $0< n_{1} \le n_{2}$

29 $$\begin{aligned} \sum _{\begin{array}{c} j \ge 1 \, , \, k \ge 0 \\ n_{1} \le j + k \le n_{2} \end{array}} \, e^{4 \Omega _{0}(j+k)} \, \Omega _{j} \, \Omega _{k}^{*}(4) \, \le \, \frac{1}{4} \, \sum _{n_{1} \le m \le n_{2}} m \, e^{4 \Omega _{0} m} \, \Omega _{m}^{*}(4). \end{aligned}$$

Let us define $U_{0}:=U_{J_{0}}$ and $U_{j}:= U_{J_{j}} - U_{J_{j-1}}$ for $j \ge 1$. Note that $U_{j}$ which has support in $J_{j}$ and satisfies the bound

30 $$\begin{aligned} \Vert U_{j} \Vert \, \le \, 2 \, \Omega _{j+1}. \end{aligned}$$

Having (29) and (30) in mind, we get by Theorem 2.3 that for $|s| \le 1$

$$\begin{aligned} \Vert \Gamma _{H_{J_{p}}}^{s}(U_{J_{p}})\Vert \le \sum _{j = 0}^{p}{\Vert \Gamma _{H_{J_{p}}}^{s}(U_{j})\Vert } \,&\le \, \sum _{j = 0}^{p} \Vert U_{j}\Vert \, e^{2 \Omega _{0} |J_{j}|} \, \left( \sum _{k = 0}^{p-j}e^{4\Omega _{0} k} \, \Omega _{k}^{*}(4) \right) \\&\le \sum _{j = 0}^{p} 2 \, \Omega _{j+1} \, e^{4 \Omega _{0} (j+1)} \, \left( \sum _{k = 0}^{p-j}e^{4 \Omega _{0} k} \, \Omega _{k}^{*}(4) \right) \\&\le \frac{1}{2} \, \sum _{m =1}^{p+1} m\, e^{4 \Omega _{0} m} \, \Omega _{m}^{*}(4). \end{aligned}$$

To prove the second bound, we can decompose

$$\begin{aligned} \Vert \Gamma _{H_{J_{p}}}^{s}(U_{J_{p}}) - \Gamma _{H_{J_{q}}}^{s}(U_{J_{q}}) \Vert&\le \, \sum _{j=p+1}^{q} \Vert \Gamma ^{s}_{H_{J_{q}}}(U_{j})\Vert \\&\quad + \sum _{j=0}^{p}{\Vert \Gamma _{H_{J_{p}}}^{s}(U_{j}) - \Gamma _{H_{J_{q}}}^{s}(U_{j})\Vert }. \end{aligned}$$

We can now bound each term separately making use of Theorem 2.3,

$$\begin{aligned} \sum _{j=0}^{p}{\Vert \Gamma _{H_{J_{p}}}^{s}(U_{j}) - \Gamma _{H_{J_{q}}}^{s}(U_{j})\Vert }\,&\, \le \, \sum _{j=0}^{p} e^{2 \Omega _{0}|J_{j}|} \, \Vert U_{j}\Vert \,\left( \sum _{k = p-j+1}^{q-j} e^{4 \Omega _{0} k} \, \Omega _{k}^{*}(4)\right) \\ \,&\, \le \, \sum _{j=0}^{p} e^{4 \Omega _{0} (j+1)} \, 2 \, \Omega _{j+1} \,\left( \sum _{k = p-j+1}^{q-j} e^{4 \Omega _{0} k} \, \Omega _{k}^{*}(4)\right) \\ \,&\, \le \, \frac{1}{2} \sum _{m = p+2}^{q+1} m \, e^{4 \Omega _{0} m} \, \Omega _{m}^{*}(4)\, \end{aligned}$$

and

$$\begin{aligned} \sum _{j=p+1}^{q}{\Vert \Gamma ^{s}_{H_{J_{q}}}(U_{j})\Vert }&\, \le \, \sum _{j=p+1}^{q} e^{2 \Omega _{0} |J_{j}|} \, \Vert Q_{j}\Vert \, \left( \sum _{k = 0}^{q-j}{e^{4 \Omega _{0} k} \, \Omega _{k}^{*}(4)}\right) \\&\le \sum _{j=p+1}^{q} e^{4 \Omega _{0} (j+1)} \, 2 \, \Omega _{j+1} \, \left( \sum _{k =0}^{q-j}{e^{4 \Omega _{0} k} \, \Omega _{k}^{*}(4)}\right) \\&\le \frac{1}{2} \sum _{m = p+2}^{q+1} m \, e^{4 \Omega _{0} m} \, \Omega _{m}^{*}(4). \end{aligned}$$

Combining both inequalities we conclude the result. $\square$

### Proof of Theorem 3.1

We just explain the argument for $E_{a,p}^{1}$ since it is analogous for the inverse. Using (26) for $E_{a,p}^{1}$ and applying (27) to each factor of (25)

$$\begin{aligned} \Vert E_{a,p}^{1}\Vert \,&\le \, \sum _{m=0}^{\infty }{ \left( \frac{1}{2} \, \sum _{k=1}^{p+1} k \, e^{4 \Omega _{0} k} \, \Omega ^{*}_{k}(4 ) \right) ^{m} } \frac{1}{m!}\, = \, \exp {\left( \frac{1}{2} \, \sum _{k =1}^{p+1}{ k \, e^{4 \Omega _{0} k} \, \Omega ^{*}_{k}(4)} \right) }. \end{aligned}$$

On the other hand, notice that for every $0 \le \beta _{1}, \ldots , \beta _{m} \le 1$ we have applying (27) and (28) that

$$\begin{aligned} \Big \Vert \prod _{j=1}^{m}&{ \Gamma _{H_{J_{p}}}^{\beta _{j}}(U_{J_{p}})} - \prod _{j=1}^{m}{\Gamma _{H_{J_{q}}}^{\beta _{j}}}(U_{J_{q}}) \Big \Vert \\&\, \le \, \sum _{k=1}^{m} \, \prod _{j = 1}^{k-1}{\Vert \Gamma _{H_{J_{q}}}^{\beta _{j}}(U_{J_{q}})\Vert } \, \cdot \, \Vert \Gamma _{H_{J_{p}}}^{\beta _{j}}(U_{J_{p}}) - \Gamma _{H_{J_{q}}}^{\beta _{j}}(U_{J_{q}}) \Vert \cdot \prod _{j = k+1}^{m}{\Vert \Gamma _{H_{J_{p}}}^{\beta _{j}}(U_{J_{p}})\Vert }\\&\le m \, \left( \frac{1}{2} \, \sum _{k = p+ 2}^{q+1} k \, e^{4 \Omega _{0} k} \, \Omega _{k}^{*}(4) \right) \,\left( \frac{1}{2} \, \sum _{k = 1}^{q+1} k \, e^{4 \Omega _{0} k} \, \Omega _{k}^{*}(4) \, \right) ^{m-1}. \end{aligned}$$

Hence

$$\begin{aligned} \Vert E_{a,p}^{1} - E_{a,q}^{1}\Vert&\le \sum _{m \ge 1} \, \frac{1}{m!} \, \sup _{0 \le \beta _{1}, \ldots , \beta _{m} \le 1} \Big \Vert \prod _{j=1}^{m} { \Gamma _{H_{J_{p}}}^{\beta _{j}}(U_{J_{p}})} - \prod _{j=1}^{m}{\Gamma _{H_{J_{q}}}^{\beta _{j}}}(U_{J_{q}}) \Big \Vert \\&\le \, \frac{1}{2} \, \left( \sum _{k = p+2}^{q+1} k \, e^{4 \Omega _{0} k} \, \Omega _{k}^{*}(4) \right) \, \exp {\left( \frac{1}{2} \sum _{k = 1}^{q+1} k\, e^{4 \Omega _{0} k} \, \Omega _{k}^{*}(4) \, \right) }. \end{aligned}$$

$$\square $$

The above estimates can be extended to the case in which the interval where the expansional is supported is not split in a symmetric way, as we show in the next result which will be useful in the next section.

### Corollary 3.3

In the conditions of the previous theorem, for each $\beta > 0, a \in \mathbb {Z}$ and $p,q \ge 0$ let us define

$$\begin{aligned} E_{a,p,q}^{\beta }:= e^{-\beta H_{[a-p,a+1+q]}} e^{\beta H_{[a-p,a]} + \beta H_{[a+1, a+1+q]}}. \end{aligned}$$

Then,

31 $$\begin{aligned} \Vert E_{a,p,q}^{\beta } \Vert ,\Vert (E_{a,p,q}^{\beta })^{-1} \Vert \le \exp {\left( \, \frac{1}{2}\, \sum _{k =1}^{\infty } k \, e^{4 \Omega _{0} \beta k} \, \Omega _{k}^{*}(4 \beta ) \, \right) }, \end{aligned}$$

and if $$q' > q \ge 0$$

32 $$\begin{aligned} \Vert E_{a,p,q}^{\beta } - E_{a,p,q'}^{\beta } \Vert \le \, \left( \sum _{k = q +2}^{\infty } k \, e^{4 \Omega _{0} \beta k} \, \Omega _{k}^{*}(4 \beta ) \, \right) \, \exp {\left( \frac{1}{2} \, \sum _{k =1}^{\infty } k \, e^{4 \Omega _{0} \beta k} \, \Omega _{k}^{*}(4 \beta ) \, \right) } \end{aligned}$$

In the previous estimate we can replace $\Vert E_{a,p,q}^{\beta } - E_{a,p,q'}^{\beta } \Vert$ with $\Vert (E_{a,p,p'}^{\beta })^{-1} - (E_{a,p,q'}^{\beta }) ^{-1} \Vert$.

### Proof

To check (31), let us consider a new interaction $\widetilde{\Phi }$ defined by $\widetilde{\Phi }_{X} = \Phi _{X}$ for each $X \subset [a-p, a+1+p']$ and $\widetilde{\Phi }_{X}=0$ otherwise. We can then rewrite the given expansional in terms of this new interaction as

$$\begin{aligned} E^{\beta }_{a,p,q} = \widetilde{E}^{\beta }_{a,M,M} \quad \text {where} \quad M=\max {\{ p,q\}}. \end{aligned}$$

Since the constants of decay of $\widetilde{\Phi }$ are bounded by those of $\Phi$, namely $\widetilde{\Omega }_{k} \le \Omega _{k}$ and $\widetilde{\Omega }_{k}^{*}(x) \le \Omega _{k}(x)$, we can apply Theorem 3.1 to conclude the result.

Let us now prove (32) using a similar strategy. We will distinguish two cases.

*Case 1*: if $q \ge p$, then we define a new interaction $\widetilde{\Phi }$ by $\widetilde{\Phi }_{X} = \Phi _{X}$ for each $X \subset [a-p, a+1+q]$ and $\widetilde{\Phi }_{X}=0$ otherwise. Since

$$\begin{aligned} E^{\beta }_{a,p,q} = \widetilde{E}^{\beta }_{a,q,q} \quad \text {and } \quad E^{\beta }_{a,p,q'} = \widetilde{E}^{\beta }_{a,q',q'}, \end{aligned}$$

we can argue as above and apply Theorem 3.1 to get the desired bounds.

*Case 2*: if $q<p$ we are going to split

$$\begin{aligned} \Vert E_{a,p,q}^{\beta } - E_{a,p,q'}^{\beta } \Vert \le \Vert E_{a,p,q}^{\beta } - E_{a,q,q}^{\beta } \Vert + \Vert E_{a,q,q}^{\beta } - E_{a,p,q'}^{\beta } \Vert . \end{aligned}$$

To upper bound the first summand, we consider a new interaction $\widetilde{\Phi }$ with $\widetilde{\Phi }_{X} = \Phi _{X}$ whenever $X \subset [a-p,a+1+q]$ and $\widetilde{\Phi }_{X}=0$ otherwise, which satisfies

$$\begin{aligned} E^{\beta }_{a,p,q} = \widetilde{E}^{\beta }_{a,p,p} \quad \text {and } \quad E^{\beta }_{a,q,q} = \widetilde{E}^{\beta }_{a,q,q}\,; \end{aligned}$$

and for the second summand we define another interaction $\widehat{\Phi }$ as $\widehat{\Phi }_{X} = \Phi _{X}$ whenever $X \subset [a-p,a+1+q']$ and $\widehat{\Phi }_{X} = 0$ otherwise, which satisfies

$$\begin{aligned} E^{\beta }_{a,q,q} = \widehat{E}^{\beta }_{a,q,q} \quad \text {and} \quad E^{\beta }_{a,p,q'} = \widehat{E}^{\beta }_{a,M,M}, \quad \text {where}\quad M=\max {\{ p, q'\}} . \end{aligned}$$

Thus,

$$\begin{aligned} \Vert E_{a,p,q}^{\beta } - E_{a,p,q'}^{\beta } \Vert \le \Vert \widetilde{E}^{\beta }_{a,p,p} - \widetilde{E}^{\beta }_{a,q,q} \Vert + \Vert \widehat{E}^{\beta }_{a,q,q} - \widehat{E}^{\beta }_{a,M,M} \Vert , \end{aligned}$$

and applying Theorem 3.1 to both summands we conclude the result. $\square$

## Phase Transitions and Thermal States

Broadly speaking, phase transitions are singularities in correlation and thermodynamic functions of the temperature or the interactions in the thermodynamic limit. It is folklore that there is no thermal phase transition in one-dimension. To be more formal, let us restrict to interactions $\Phi$ on the one-dimensional lattice $\mathbb {Z}$. For each interval $[a,b] \subset \mathbb {Z}$ the *local Gibbs state on* [*a*, *b*] *at inverse temperature* $\beta$ is defined as

$$\begin{aligned} \varphi _{\beta }^{[a,b]}(Q) = \frac{{\textrm{tr}}(e^{- \beta H_{[a,b]}} Q)}{{\textrm{tr}}(e^{- \beta H_{[a,b]}})}, \quad Q \in \mathcal {A}_{\mathbb {Z}} . \end{aligned}$$

For general C$^*$-dynamical systems, equilibrium states at inverse temperature $\beta$ are defined in terms of the KMS condition and may feature subtle issues such as non-existence and non-uniqueness. Indeed, the existence and uniqueness of these KMS states is a feature of the absence of phase transition. These properties are satisfied in our setting for rather general conditions on the interaction, see [7]. In classical systems this is a result by Ruelle [43], while in quantum systems there are uniqueness results by Araki [3] and Kishimoto [23] (see also [8, Section 6.2.5]) that apply to interactions with uniformly bounded surface energies and, in particular, to bounded interactions $\Phi$ on $\mathbb {Z}$. They yield that for every $\beta > 0$ the following limit exists

$$\begin{aligned} \varphi _{\beta }(Q) = \lim _{\begin{array}{c} a \rightarrow - \infty \\ b \rightarrow \infty \end{array}}{\varphi _{\beta }^{[a,b]}(Q)}, \quad Q \in \mathcal {A}. \end{aligned}$$

and defines the unique equilibrium state at inverse temperature $\beta$.

Another feature of phase transitions is the clustering property of $\varphi _{\beta }$, namely the decay of the correlation function

$$\begin{aligned} {\text {Corr}}_{\beta }(Q_{1},Q_{2}) = \varphi _{\beta }(Q_{1}Q_{2}) - \varphi _{\beta }(Q_{1}) \varphi _{\beta }(Q_{2}). \end{aligned}$$

in terms of the distance that separates the support of the observables $Q_{1}$ and $Q_{2}$. That these correlations decay exponentially fast can be proven for rather general graphs at sufficiently high temperatures (small $\beta$) under suitable conditions on the interactions by means of cluster expansion techniques, see e.g. Kliesch et al. [24] in the finite range case, or the more recent result by Kuwahara et al. [26] for the decay of the conditional mutual information even under long-range interactions. Similar ideas have been also applied to prove analyticity of thermodynamic and correlation functions at high enough temperatures, see [11, Chapter III].

For low temperatures, it seems however that the typical arguments rely on the *transfer operator method*. The idea is to construct for the given interaction $\Phi$ an operator $\mathcal {L}^{\Phi }$ on the space of observables. If $\Phi$ decays sufficiently fast, then $\mathcal {L}^{\Phi }$ has “nice” spectral properties in the spirit of Perron–Frobenius theorem, that in turn yield “nice” properties for our thermodynamic and correlation functions, see [4, 30, 45].

In classical lattice systems, this approach was exploited by Ruelle [43] to show that, under suitable polynomial decay assumptions on the interaction, correlations decay exponentially fast at every temperature. Moreover, Araki [1] showed that the pressure and correlation functions depend analytically on the temperature and the interaction if the latter decay exponentially fast, see also [45]. This result was later improved by Dobrushin [10] to more general interactions. Remark that there are examples of two-body interactions with polynomial decay in one-dimension featuring phase transition, see Dyson [12]. We refer to [11] and references therein for further information.

In quantum lattice systems, however, it seems that the only known result for low temperatures is due to Araki [1] for finite range interactions. Using the locality estimates from previous sections we extend Araki’s result in the following form.

### Theorem 4.1

Let us consider a quantum spin system over $\mathbb {Z}$ and a translationally invariant interaction $\Phi$ with exponential decay $\Vert \Phi \Vert _{\lambda }<\infty$ for some $\lambda > 0$. Then, for each $\beta \in (0, \frac{\lambda }{2 \Omega _{0}})$ there exist $C,\delta > 0$ satisfying:

(i) For every $a,b \in \mathbb {Z}$ with $a < b$ and every $Q \in \mathcal {A}_{[a,b]}$
  $$\begin{aligned} |\varphi _{\beta }(Q) - \varphi _{\beta }^{[a-k,b+k]}(Q)| \le C e^{- \delta k}. \end{aligned}$$
(ii) For every $x \in \mathbb {Z}$ and $k \in \mathbb {N}$, if $Q_{1} \in \mathcal {A}_{(- \infty , x]}$ and $Q_{2} \in \mathcal {A}_{[x+k, \infty )}$ then
  $$\begin{aligned} |\varphi _{\beta }(Q_{1}Q_{2}) - \varphi _{\beta }(Q_{1}) \, \varphi _{\beta }(Q_{2})| \, \le \, C e^{ - \delta k}. \end{aligned}$$
  In other words, $\varphi _{\beta }$ has exponential decay of correlations.

Let us stress that a main feature of the given threshold $\frac{\lambda }{2 \Omega _{0}}$ is that it grows to infinity as $\lambda$ grows to infinity. Thus, in particular, if the interactions decay faster than any exponential then correlations decay exponentially fast for all temperatures. This should be compared with other high-temperature estimates for exponentially-decaying interactions. For instance, Fröhlich and Uelstchi [13] consider a local interaction $\Phi$ on $\mathbb {Z}^{g}$ and a nonnegative function *b*(*X*) over the finite subsets of $\mathbb {Z}^{g}$, and prove that if $a>0$ and $\beta >0$ satisfy

$$\begin{aligned} \beta \, \sup _{x \in \mathbb {Z}^{g}} \, \sum _{X \ni x} \Vert \Phi _{X}\Vert \, e^{\frac{3}{2}a|X| + b(X)} < a, \end{aligned}$$

then we have exponential decay of correlations at (inverse) temperature $\beta$. Observe that the above condition yields

$$\begin{aligned} \beta \, e^{\frac{3}{2}a} \Omega _{0} \, \le \, \beta \, \sup _{x \in \mathbb {Z}^{g}} \, e^{\frac{3}{2}a}\sum _{X \ni x} \Vert \Phi _{X}\Vert < a , \end{aligned}$$

so their result gives exponential decay of correlations for a range of values of $\beta$ which is uniformly bounded, since

$$\begin{aligned} \beta \le \frac{a}{e^{\frac{3}{2} a} \, \Omega _{0}} \le \frac{1}{\Omega _{0}}. \end{aligned}$$

This result has, of course, a clear advantage as opposed to the above theorem, namely that it holds for dimensions larger than one.

### Idea of the proof: notation

The proof follows the lines of the original argument by Araki [1], its later extension by Golodets and Neshveyev [14] to AF-algebras, and the more recent work on the non-commutative Ruelle transfer operator by Matsui [30].

We can absorb $\beta$ in the interaction, and so we just have to prove the case $\beta = 1$ of the theorem assuming that

$$\begin{aligned} \lambda > 2 \Omega _{0}. \end{aligned}$$

We analyze the one-sided version of the problem on $\mathcal {A}_{\mathbb {N}}$. Let us consider for every $a \ge 1$

$$\begin{aligned} \varphi ^{[1,a]}\left( Q \right) = \frac{{\textrm{tr}}\left( e^{-H_{[1,a]}}Q \right) }{{\textrm{tr}}\left( e^{-H_{[1,a]}} \right) }, \quad Q \in \mathcal {A}_{\mathbb {N}}. \end{aligned}$$

Fixed $1 \le n < a$ let us denote

$$\begin{aligned} \widetilde{E}_{(n,a)}&:=e^{-\frac{1}{2}H_{[1,a]}} e^{\frac{1}{2} H_{[1+n,a]}}\\ E_{(n,a)}&:=e^{-\frac{1}{2}H_{[1,a]}} e^{\frac{1}{2}H_{[1,n]} + \frac{1}{2} H_{[1+n,a]}} \end{aligned}$$

which satisfy

33 $$\begin{aligned} e^{-\frac{1}{2}H_{[1,a]}} \, = \, \widetilde{E}_{(n,a)} \, e^{-\frac{1}{2}H_{[1+n,a]}} \, = \, E_{(n,a)} \, e^{-\frac{1}{2}H_{[1,n]} - \frac{1}{2} H_{[1+n,a]}}. \end{aligned}$$

**Fig. 1:**
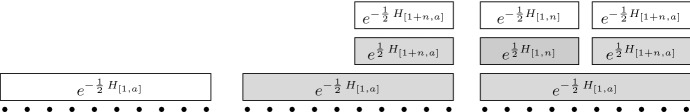
From left to right, decomposition of $e^{-\frac{1}{2}H_{[1,a]}}$ in terms of the factors $\widetilde{E}_{(n,a)}$ and $E_{(n,a)}$ represented both with shaded boxes

Using that $\tau _{-n}$ is an algebra homomorphism from $\mathcal {A}_{[1+n,\infty )}$ into $\mathcal {A}_{[1,\infty )}$, we have

$$\begin{aligned} {\textrm{tr}}\left( e^{-H_{[1,a]}}Q\right)&\, = \, {\textrm{tr}}\big ( \,e^{-H_{[1+n,a]}} \, \widetilde{E}_{(n,a)}^{\dagger } \,Q \, \widetilde{E}_{(n,a)} \, \big )\\&\, = \, {\textrm{tr}}\left( \, e^{-H_{[1,a-n]}} \, \mathcal {L}_{(n,a)}(Q)\, \right) , \end{aligned}$$

where $\mathcal {L}_{(n,a)}:\mathcal {A}_{\mathbb {N}} \longrightarrow \mathcal {A}_{\mathbb {N}}$ is given by

$$\begin{aligned} \mathcal {L}_{(n,a)}(Q) := \tau _{-n} {\textrm{tr}}_{[1,n]}\big (\widetilde{E}_{(n,a)}^{\dagger } Q \widetilde{E}_{(n,a)} \big )\, = \, \tau _{-n} {\textrm{tr}}_{[1,n]}\big (e^{-H_{[1,n]}}\, E_{(n,a)}^{\dagger } Q E_{(n,a)} \big ). \end{aligned}$$

Therefore, we can rewrite the local Gibbs state as

34 $$\begin{aligned} \varphi ^{[1,a]}(Q) \, = \, \frac{\varphi ^{[1,a-n]}\big ( \mathcal {L}_{(n,a)}(Q)\big )}{\varphi ^{[1,a-n]}\big ( \mathcal {L}_{(n,a)}(\mathbb {1})\big )}. \end{aligned}$$

The rest of the proof has been decomposed into several stages corresponding to the following subsections. Let us briefly sketch them here:

1. The estimates on the expansionals given in Sect. 3 yield that for every $n \in \mathbb {N}$ the elements $E_{(n,a)}$ converge as $a \rightarrow + \infty$ to a certain $E_{n}$ exponentially fast. In particular, the family $\mathcal {L}_{(n,a)}$ strongly converges to some operator $\mathcal {L}_{n}$.
2. The sequence $\mathcal {L}_{n}$ satisfies $\mathcal {L}_{n} = \mathcal {L} \circ \cdots \circ \mathcal {L}$ (*n* times) where $\mathcal {L} : = \mathcal {L}_{1}$ is the so-called *Ruelle transfer operator* that maps $\mathcal {A}_{\mathbb {N}}(x)$ into $\mathcal {A}_{\mathbb {N}}(x)$ for a suitable constant $x>1$ (recall Sect. 1.1 for notation). Moreover, there exist a positive constant $\mu >0$, a state $\nu$ over $\mathcal {A}_{\mathbb {N}}$ and a positive and invertible observable *h* in $\mathcal {A}_{\mathbb {N}}(x)$ such that the rank-one operator
  $$\begin{aligned} \mathcal {P}:\mathcal {A}_{\mathbb {N}}(x) \longrightarrow \mathcal {A}_{\mathbb {N}}(x) , \quad Q \,\, \longmapsto \,\, \nu (Q) h \end{aligned}$$
  satisfies
  (i) $$\mathcal {P} \circ \mathcal {P} = \mathcal {P}$$,
  (ii) $$\mathcal {L} \circ \mathcal {P} = \mathcal {P} \circ \mathcal {L} = \mu \, \mathcal {P}$$.
  In particular, we can decompose for every $n \in \mathbb {N}$
  $$\begin{aligned} \mathcal {L}^{n} = \mu ^{n} \, \mathcal {P} \, + \, \mathcal {L}^{n}(\mathbb {1} - \mathcal {P}). \end{aligned}$$
3. The rescaled operator $L := \mu ^{-1} \mathcal {L}: \mathcal {A}_{\mathbb {N}}(x) \longrightarrow \mathcal {A}_{\mathbb {N}}(x)$ satisfies that there is $\gamma >1$ such that
  $$\begin{aligned} \lim _{n \rightarrow \infty }{\gamma ^{n}} \, {\left| \left| \left| L^{n}(\mathbb {1} - \mathcal {P}) \right| \right| \right| }_{1,x} = 0. \end{aligned}$$
4. The sequence $\nu \circ \tau _{n}$ converges to a translation invariant state $\psi ^{[1,\infty )}$ on $\mathcal {A}_{[1,\infty )}$, which can be extended to a (unique) translation invariant state $\psi$ over $\mathcal {A}_{\mathbb {Z}}$ satisfying exponential decay of correlations. The state $\psi$ is actually the infinite-volume KMS state associated to $\Phi$ at $\beta =1$.

### The map $\mathcal {L}_{n}$: definition and basic properties

We have to prove that, fixed *n*, $E_{(n,a)}$ and $\widetilde{E}_{(n,a)}$ converge when *a* tends to infinity. For that we will make use of Theorem 3.1 and its subsequent Corollary 3.3. Let us consider the next slightly modified version of the error terms that appear there:

35 $$\begin{aligned} \begin{aligned} \mathcal {G}&:= \exp {\left( \, {\sum }_{k \ge 1} k \, e^{2 \Omega _{0} k} \, \Omega _{k}^{*}(2) \, \right) } \, , \\ \mathcal {G}_{\ell }&:= \mathcal {G} \cdot {\sum }_{k \ge \ell } k \, e^{2 \Omega _{0} k} \, \Omega _{k}^{*}(2)\quad (\ell \ge 1). \end{aligned} \end{aligned}$$

As a consequence of the hypothesis $\lambda >2 \Omega _{0}$, we have that $\mathcal {G}$ is finite and $\mathcal {G}_{\ell }$ decays exponentially fast. Indeed, using (19) we can estimate for every $\ell \ge 1$

$$\begin{aligned} \sum _{k \ge \ell } k e^{2 \Omega _{0} k} \Omega _{k}^{*}(2) = \sum _{k \ge \ell } k e^{(2 \Omega _{0}- \lambda ) k} e^{\lambda k} \Omega _{k}^{*}(2) \le \big ( \max _{k \ge \ell } k e^{(2 \Omega _{0} - \lambda )k}\big ) e^{2 \Vert \Phi \Vert _{\lambda }} < \infty . \end{aligned}$$

We then have the following convergence result.

#### Proposition 4.2

For each $n \in \mathbb {N}$ the following limits exist and are invertible

$$\begin{aligned} E_{n} := \lim _{a \rightarrow + \infty }E_{(n,a)}, \quad \widetilde{E}_{n} := \lim _{a \rightarrow + \infty }\widetilde{E}_{(n,a)} = E_{n} \, e^{-\frac{1}{2}H_{[1,n]}}. \end{aligned}$$

Moreover, they satisfy for every $1 \le n < a$:

(i) $$\Vert E_{n}\Vert , \Vert E_{(n,a)}\Vert , \Vert E_{n}^{-1}\Vert , \Vert E_{(n,a)}^{-1}\Vert \le \mathcal {G}$$,
(ii) $$\Vert E_{(n,a)} - E_{n} \Vert , \Vert E_{(n,a)}^{-1} - E_{n}^{-1} \Vert \le \mathcal {G}_{a-n}$$,
(iii) For every positive and invertible $Q \in \mathcal {A}_{\mathbb {N}}$ and $E \in \{ E_{(n,a)}, E_{n} \}$
  $$\begin{aligned} \mathcal {G}^{-2} \, \Vert Q^{-1}\Vert ^{-1} \,\mathbb {1} \, \le \, E^{\dagger } Q E \, \le \, \mathcal {G}^{2} \, \Vert Q\Vert \, \mathbb {1}. \end{aligned}$$

#### Proof

Item (*iii*) follows from (*i*). Applying Corollary 3.3, we deduce that for every $n, a,a' \in \mathbb {N}$ with $n<a \le a'$

36 $$\begin{aligned} \Vert E_{(n,a)}\Vert , \Vert E_{(n,a)}^{-1}\Vert \le \mathcal {G} \quad \text {and} \quad \Vert E_{(n,a)}^{-1} - E_{(n,a')}^{-1} \Vert , \Vert E_{(n,a)} - E_{(n,a')} \Vert \le \mathcal {G}_{a-n}. \end{aligned}$$

The last Cauchy condition yields that, fixed *n*, $E_{(n,a)}$ converges to some $E_{n}$ when *a* tends to infinity. Analogously, $E_{(n,a)}^{-1}$ also converges, so its limit must be equal to $E_{n}^{-1}$. Leaving *a* fixed and taking limit when $a'$ tends to infinity on the right-hand side of (36) we obtain (*ii*). On the other hand, taking limit in *a* on the left-hand side of (36) leads to the remaining inequalities of (*i*) involving $E_{n}$ and its inverse. $\square$

#### Definition 4.3

For every $n \in \mathbb {N}$, let $\mathcal {L}_{n}:\mathcal {A}_{\mathbb {N}} \longrightarrow \mathcal {A}_{\mathbb {N}}$ be the positive linear operator

$$\begin{aligned} \mathcal {L}_{n}(Q) := \tau _{-n} \, {\textrm{tr}}_{[1,n]}\Big ( e^{- H_{[1, n]} } \, E_{n}^{\dagger }\, Q \, E_{n} \Big ) \, = \, \tau _{-n} \, {\textrm{tr}}_{[1,n]}\Big ( \widetilde{E}_{n}^{\dagger }\, Q \, \widetilde{E}_{n} \Big ) . \end{aligned}$$

We will simply denote $\mathcal {L}:= \mathcal {L}_{1}$.

#### Theorem 4.4

Let $n, \ell \in \mathbb {N}$ and $Q,A \in \mathcal {A}_{\mathbb {N}}$. Then,

(i) $$\Vert \mathcal {L}_{n}(Q)\Vert \le {\textrm{tr}}(e^{-H_{[1,n]}}) \, \mathcal {G}^{2} \, \Vert Q\Vert $$,
(ii) $$\Vert \mathcal {L}_{n}(Q) - \mathcal {L}_{(n,n+\ell )}(A)\Vert \, \le \, {\textrm{tr}}(e^{-H_{[1,n]}}) \, \big (\, 2 \mathcal {G} \, \mathcal {G}_{\ell } \Vert Q\Vert + \mathcal {G}^{2} \, \Vert Q - A\Vert \big )$$,
(iii) $$\Vert \mathcal {L}_{n}(Q)\Vert _{\ell } \le {\textrm{tr}}(e^{-H_{[1,n]}}) \, (2 \mathcal {G} \mathcal {G}_{\ell } \Vert Q\Vert + \mathcal {G}^{2}\Vert Q\Vert _{n+\ell })$$.

Moreover, if $Q \in \mathcal {A}_{\mathbb {N}}$ is positive and invertible, then

(iv) $${\textrm{tr}}(e^{-H_{[1,n]}}) \, \mathcal {G}^{-2} \, \Vert Q^{-1}\Vert ^{-1} \, \mathbb {1}\, \le \, \mathcal {L}_{n}(Q) \, \le \,{\textrm{tr}}(e^{-H_{[1,n]}}) \, \mathcal {G}^{2} \, \Vert Q\Vert \, \mathbb {1}$$,
(v) $$ \Vert \mathcal {L}_{n}(Q)\Vert _{\ell } \, \Vert \mathcal {L}_{n}(Q)^{-1}\Vert \, \le \, 2 \, \mathcal {G}^{3} \, \mathcal {G}_{\ell } \, \Vert Q\Vert \, \Vert Q^{-1}\Vert + \mathcal {G}^{4} \, \Vert Q\Vert _{n+\ell } \, \Vert Q^{-1} \Vert $$.

#### Proof

All statements are consequence of Proposition 4.2. Items (*i*) and (*iv*) are straightforward. Let us check that (*ii*) holds.

$$\begin{aligned} \Vert \, \mathcal {L}_{n}(Q) - \mathcal {L}_{(n,n+\ell )}(A) \, \Vert \, \le \, {\textrm{tr}}\big ( e^{-H_{[1, n]}}\big ) \, \Vert E_{n}^{\dagger } Q E_{n} - E_{(n,n+\ell )}^{\dagger } A E_{(n,n+\ell )}^{\dagger }\Vert , \end{aligned}$$

where

$$\begin{aligned} \begin{aligned} \Vert E_{n}^{\dagger } Q E_{n} - E_{(n,n+\ell )}^{\dagger } A E_{(n,n+\ell )} \Vert&\le \Vert E_{n}^{\dagger } Q E_{n} - E_{(n,n+\ell )}^{\dagger } Q E_{(n,n+\ell )} \Vert + \mathcal {G}^{2} \, \Vert Q - A\Vert \\&\le 2 \, \mathcal {G} \, \mathcal {G}_{\ell } \, \Vert Q\Vert + \mathcal {G}^{2} \Vert Q - A \Vert . \end{aligned} \end{aligned}$$

To prove (*iii*), let us take $\widetilde{Q} \in \mathcal {A}_{[1,n+\ell ]}$ satisfying $\Vert Q - \widetilde{Q}\Vert = \Vert Q\Vert _{n+\ell }$. Then, $\mathcal {L}_{(n, n+\ell )}(\widetilde{Q})$ belongs to $\mathcal {A}_{[1, \ell ]}$, and so by (*ii*)

$$\begin{aligned} \Vert \mathcal {L}_{n}(Q)\Vert _{\ell } \, \le \, \Vert \mathcal {L}_{n}(Q) - \mathcal {L}_{(n,n+\ell )}(\widetilde{Q}) \Vert \, \le \, {\textrm{tr}}(e^{-H_{[1,n]}}) \, \big (\, 2 \, \mathcal {G} \, \mathcal {G}_{\ell } \, \Vert Q\Vert + \mathcal {G}^{2} \, \Vert Q\Vert _{n+\ell } \big ). \end{aligned}$$

Finally, combining (*iii*) and (*iv*) we conclude that (*v*) holds. $\square$

A consequence of the previous theorem is that for every positive and invertible element $Q \in \mathcal {A}_{\mathbb {N}}$ it holds $\Vert \mathcal {L}_{n}(Q)\Vert \, \Vert \mathcal {L}_{n}(Q)^{-1} \Vert \le \mathcal {G}^{4} \Vert Q\Vert \, \Vert Q^{-1}\Vert$. The next result improves this estimate for large values of *n*.

#### Theorem 4.5

Let $Q \in \mathcal {A}_{\mathbb {N}}$ be positive and invertible and let $n, \ell \in \mathbb {N}$ such that $1 + \ell \le n$. Then

$$\begin{aligned} \Vert \mathcal {L}_{n}(Q)\Vert \Vert \mathcal {L}_{n}(Q)^{-1}\Vert \,&\le \, \mathcal {G}^{4} \left( 1 + 4 \, \mathcal {G}^{3} \, \mathcal {G}_{\ell } \, \Vert Q\Vert \, \Vert Q^{-1}\Vert \, + 4 \, \mathcal {G}^{4} \, \Vert Q\Vert _{n-\ell }\, \Vert Q^{-1}\Vert \right) . \end{aligned}$$

#### Proof

Let us write

$$\begin{aligned} E_{(n,n+\ell )}' \, := \, e^{-\frac{1}{2}H_{[n-\ell +1, n+\ell ]}} \, e^{\frac{1}{2}H_{[n-\ell +1, n]}} \, e^{\frac{1}{2} H_{[1+n,n+\ell ]}},, \end{aligned}$$

whose support is contained in $[ n - \ell +1, n + \ell ]$. Note that Corollary 3.3 yields

37 $$\begin{aligned} \Vert E_{(n,n+\ell )}'\Vert \le \mathcal {G} \quad \text {and} \quad \Vert E_{(n,n+\ell )}' - E_{(n,n+\ell )} \Vert \le \mathcal {G}_{\ell }. \end{aligned}$$

Using the tracial state over $\mathcal {A}_{[n-\ell +1, \infty )}$, let us define

$$\begin{aligned} A := {\textrm{tr}}_{[ n-\ell +1, \infty )} (Q) \in \mathcal {A}_{[1,n-\ell ]} \end{aligned}$$

which is positive and invertible. Notice that *A* and $E_{(n,n+\ell )}'$ have disjoint support and thus

$$\begin{aligned} \widetilde{A} \,&:= \tau _{-n} \, {\textrm{tr}}_{[1,n]}\big ( e^{-H_{[1,n]} } E_{(n,n+\ell )}'^{\, \dagger } \, A \, E_{(n,n+\ell )}' \big )\\&= \tau _{-n} \, {\textrm{tr}}_{[1,n]}\big ( e^{-H_{[1,n]} } A^{1/2} E_{(n,n+\ell )}'^{\, \dagger } E_{(n,n+\ell )}' A^{1/2} \big ) . \end{aligned}$$

Consequently, applying (37) we get

38 $$\begin{aligned} \mathcal {G}^{-2} \, {\textrm{tr}}\big ( e^{- H_{[1,n]}} A \big ) \mathbb {1} \, \le \, \widetilde{A} \,\le \, \mathcal {G}^{2} \, {\textrm{tr}}\big ( e^{- H_{[1,n]}} A \big ) \mathbb {1}. \end{aligned}$$

Let $\phi$ and $\phi '$ be two states over $\mathcal {A}_{\mathbb {N}}$. From (38) it follows that

$$\begin{aligned} \phi (\widetilde{A}) \, \le \, \mathcal {G}^{4} \, \phi '(\widetilde{A})\, \end{aligned}$$

and thus

$$\begin{aligned} \phi (\mathcal {L}_{n}(Q)) \,&\le \, \phi (\widetilde{A}) + \Vert \, \mathcal {L}_{n}(Q) - \widetilde{A} \, \Vert \\&\, \le \, \mathcal {G}^{4} \, \phi '(\widetilde{A}) + \Vert \, \mathcal {L}_{n}(Q)- \widetilde{A} \, \Vert \\&\, \le \, \mathcal {G}^{4} \, \phi '(\mathcal {L}_{n}(Q)) + (1 + \mathcal {G}^{4}) \, \Vert \, \mathcal {L}_{n}(Q) - \widetilde{A} \, \Vert . \end{aligned}$$

Using that for every positive and invertible $B \in \mathcal {A}_{\mathbb {N}}$

$$\begin{aligned} \Vert B\Vert = \sup _{\phi }{\phi (B)} \quad \quad \text{ and } \quad \quad \Vert B^{-1}\Vert ^{-1} = \inf _{\phi '}{\phi '(B)}, \end{aligned}$$

where the supremum and infimum are both taken with respect to all states over $\mathcal {A}_{\mathbb {N}}$, we conclude that

39 $$\begin{aligned} \begin{aligned} \Vert \mathcal {L}_{n}(Q) \Vert \, \Vert \mathcal {L}_{n}(Q)^{-1}\Vert \,&\le \, \mathcal {G}^{4} + (1 + \mathcal {G}^{4}) \, \Vert \mathcal {L}_{n}(Q) - \widetilde{A} \Vert \, \Vert \mathcal {L}_{n}(Q)^{-1}\Vert \\&\le \mathcal {G}^{4} \, \left( 1 + 2 \, \Vert \mathcal {L}_{n}(Q) - \widetilde{A} \Vert \, \Vert \mathcal {L}_{n}(Q)^{-1}\Vert \right) . \end{aligned} \end{aligned}$$

Observe that But notice that the same argument given in the proof of Theorem 4.4.(*ii*) but now using (37) leads to

$$\begin{aligned} \Vert \mathcal {L}_{n}(Q) - \widetilde{A} \Vert \le \, {\textrm{tr}}(e^{-H_{[1,n]}}) \, \big (\, 2 \, \mathcal {G} \, \mathcal {G}_{\ell } \, \Vert Q\Vert + \mathcal {G}^{2} \, \Vert Q - A\Vert \big ), \end{aligned}$$

and thus using Theorem 4.4.(*iv*)

40 $$\begin{aligned} \Vert \mathcal {L}_{n}(Q) - \widetilde{A}\Vert \, \Vert \mathcal {L}_{n}(Q)^{-1}\Vert \, \le \, 2 \, \mathcal {G}^{3} \, \mathcal {G}_{\ell } \, \Vert Q\Vert \, \Vert Q^{-1}\Vert \, + \mathcal {G}^{4} \, \Vert Q - A\Vert \, \Vert Q^{-1}\Vert . \end{aligned}$$

To upper bound the last summand of (40), we use that taking $Q_{n- \ell } \in \mathcal {A}_{[1,n-\ell ]}$ with $\Vert Q - Q_{n -\ell }\Vert =\Vert Q\Vert _{n- \ell }$, it holds that

41 $$\begin{aligned} \Vert Q - A\Vert = \Vert Q- Q_{n -\ell } + {\textrm{tr}}_{[n - \ell +1 , \infty )} (Q_{n -\ell } - Q) \Vert \le 2 \, \Vert Q\Vert _{n- \ell }. \end{aligned}$$

Combining (39), (40) and (41), we conclude the result. $\square$

### The maps $\mathcal {L}$ and *L*: fixed points

#### Proposition 4.6

For every $$n \in \mathbb {N}$$, $$\mathcal {L}_{n} = \mathcal {L}^{n} := \mathcal {L} \circ \cdots \circ \mathcal {L} \,\,\, (n \text{ times})$$.

#### Proof

For every $1\le n < a$, it is easy to check that

$$\begin{aligned} \widetilde{E}_{(n,a+n)} \, \tau _{n}\big ( \widetilde{E}_{(1,a)} \big ) = \widetilde{E}_{(n+1, n+a)}, \end{aligned}$$

and so

$$\begin{aligned} \mathcal {L}_{(1,a)} \circ \mathcal {L}_{(n,a+n)} = \mathcal {L}_{(n+1, a+n)}. \end{aligned}$$

By Theorem 4.4, we can take limit in the previous expression when $a \rightarrow + \infty$ to conclude that $\mathcal {L}_{1} \circ \mathcal {L}_{n} = \mathcal {L}_{n+1}$. $\square$

#### Theorem 4.7

There exist a state $\nu$ over $\mathcal {A}_{\mathbb {N}}$ and a real number $\mu > 0$ such that

42 $$\begin{aligned} \nu (\mathcal {L}(Q)) = \mu \, \nu (Q) \quad \text{ for } \text{ every } Q \in \mathcal {A}_{\mathbb {N}}. \end{aligned}$$

Moreover, it satisfies that for every $n \in \mathbb {N}$

43 $$\begin{aligned} \mathcal {G}^{-2} \, {\textrm{tr}}\big ( e^{- H_{[1,n]}} \big ) \, \le \, \mu ^{n} \, \le \, \mathcal {G}^{2} \, {\textrm{tr}}\big ( e^{- H_{[1,n]}} \big ). \end{aligned}$$

#### Proof

Let us first observe that $\mathcal {L}: \mathcal {A}_{\mathbb {N}} \longrightarrow \mathcal {A}_{\mathbb {N}}$ is a positive linear operator on the C*-algebra $\mathcal {A}_{\mathbb {N}}$ satisfying that $\mathcal {L}(\mathbb {1}) \ge \gamma \mathbb {1}$ for a positive constant $\gamma > 0$, by Theorem 4.4. Its dual operator $\mathcal {L}^{*}:\mathcal {A}_{\mathbb {N}}^{*} \longrightarrow \mathcal {A}_{\mathbb {N}}^{*}$ is weak$^*$-weak$^*$ continuous, and so does its restriction $\mathcal {L}^{*} : \mathcal {S} \longrightarrow \mathcal {A}_{\mathbb {N}} ^{*}$ to the weak$^*$-compact and convex subset $\mathcal {S} \subset \mathcal {A}_{\mathbb {N}}^{*}$ of all states over a $\mathcal {A}_{\mathbb {N}}$. Next, let us define

$$\begin{aligned} \widehat{\mathcal {L}} : \mathcal {S} \longrightarrow \mathcal {S} , \quad \widehat{\mathcal {L}} (\varphi ) = \frac{\mathcal {L}^{*} \varphi }{\mathcal {L}^{*} \varphi (\mathbb {1})} \quad \text {for every}\quad \varphi \in \mathcal {S}. \end{aligned}$$

We claim that it is well-defined. Indeed, observe that for every $\varphi \in \mathcal {S}$ we have the uniform lower bound

44 $$\begin{aligned} \mathcal {L}^{*} \varphi (\mathbb {1}) = \varphi (\mathcal {L} ( \mathbb {1})) \ge \gamma \varphi (\mathbb {1}) = \gamma > 0. \end{aligned}$$

Hence the map $\widehat{\mathcal {L}}(\varphi )$ is linear and positive, since for each $Q \ge 0$ we have $\mathcal {L}^{*}\varphi (Q) = \varphi (\mathcal {L}(Q)) \ge 0$ using that $\mathcal {L}$ is positive and $\varphi$ is a state. Combining this with the fact that $\widehat{\mathcal {L}}(\varphi )(\mathbb {1}) = 1$, we conclude that $\widehat{\mathcal {L}}(\varphi ) \in \mathcal {S}$. This proves the claim.

Moreover, we have that the operator $\widehat{\mathcal {L}}$ is also $\hbox {weak}^*$–$\hbox {weak}^*$-continuous, which is a straightforward consequence of the fact that $\mathcal {L}^{*}$ is $\hbox {weak}^*$–$\hbox {weak}^*$-continuous together with the uniform lower bound (44). We are thus in the conditions to apply Schauder–Tychonov theorem (see [42, Theorem 5.28]) to the map $\widehat{\mathcal L}$ and the locally convex space $(\mathcal {A}_{\mathbb {N}}, {\text {weak}}^*)$. We deduce the existence of a fixed point $\nu \in \mathcal {S}$ of $\widehat{\mathcal {L}}$. In particular, for every $Q \in \mathcal {A}_{\mathbb {N}}$ it has to satisfy

$$\begin{aligned} \nu (\mathcal {L}(Q)) = \mu \, \nu (Q) \quad \text{ where } \quad \mu := \nu (\mathcal {L}(\mathbb {1})). \end{aligned}$$

This proves the first statement of the theorem. To check second one, we simply apply the state $\nu$ to each term in Theorem 4.4.(*iv*) with $Q = \mathbb {1}$. $\square$

#### Definition 4.8

Let us denote by $L: \mathcal {A}_{\mathbb {N}} \longrightarrow \mathcal {A}_{\mathbb {N}}$ the operator given by $L = \mathcal {L}/\mu$, where $\mu > 0$ is the positive number provided by Theorem 4.7. We will also write $L_{(n,a)} = \mathcal {L}_{(n,a)}/\mu ^{n}$ for every $a>n \ge 1$.

The next result collects some straightforward properties of *L* inherited from $\mathcal {L}$.

#### Corollary 4.9

Let $n, \ell \in \mathbb {N}$ and $Q \in \mathcal {A}_{\mathbb {N}}$. Then

(i) $$\Vert L^{n}(Q) \Vert \le \mathcal {G}^{4} \, \Vert Q\Vert $$,
(ii) $$\Vert L^{n}(Q) - L_{(n,n+\ell )}(Q)\Vert \le 2 \mathcal {G}^{3} \mathcal {G}_{\ell } \Vert Q \Vert $$,
(iii) $\Vert L^{n}(Q)\Vert _{\ell } \le 2 \mathcal {G}^{3}\mathcal {G}_{\ell } \, \Vert Q\Vert + \mathcal {G}^{4} \Vert Q\Vert _{n +\ell }$. Moreover, if *Q* is positive and invertible, then
(iv) $$\mathcal {G}^{-4} \, \Vert Q^{-1}\Vert ^{-1} \, \mathbb {1} \, \le \, L^{n}(Q) \, \le \, \mathcal {G}^{4} \, \Vert Q\Vert \, \mathbb {1}$$.

In particular, for every $x>1$ satisfying $\sum _{\ell \ge 1}{\mathcal {G}_{\ell }\, x^{\ell }} < \infty$ we have that the restriction $L: \mathcal {A}_{\mathbb {N}} (x) \longrightarrow \mathcal {A}_{\mathbb {N}} (x)$ is well-defined and continuous. Indeed, there is a constant $C_{x} > 0$ such that for every $m,n \in \mathbb {N}$ and every $Q \in \mathcal {A}_{\mathbb {N}} (x)$

$$\begin{aligned} {\left| \left| \left| L^{n}(Q) \right| \right| \right| }_{m,x} \le C_{x} \, {\left| \left| \left| Q \right| \right| \right| }_{n+m,x}. \end{aligned}$$

#### Proof

Items (i)–(iv) follow from Theorem 4.4. (i)–(iv), dividing by $\mu ^{n}$ on both sides of each inequality and using (43). To check the last statement, let $Q \in \mathcal {A}_{\mathbb {N}}(x)$ with $x > 1$ satisfying $\sum _{\ell \ge 1}{\mathcal {G}_{\ell } \, x^{\ell }}< \infty$ and let $m,n \in \mathbb {N}$. Using (i) and (iii) we can estimate

$$\begin{aligned} {\left| \left| \left| L^{n}(Q) \right| \right| \right| }_{m,x}&= \Vert L^{n}(Q)\Vert + \sum _{\ell \ge m}{\Vert L^{n}(Q)\Vert _{\ell } \, x^{\ell }}\\&\le \mathcal {G}^{4}\Vert Q\Vert + 2\, \mathcal {G}^{3} \, \Vert Q\Vert \, \sum _{\ell \ge m}{\mathcal {G}_{\ell } x^{\ell }} + \mathcal {G}^{4} \, \sum _{\ell \ge m}{\Vert Q\Vert _{n+\ell } \, x^{\ell }}\\&\le \Big ( \mathcal {G}^{4} + 2 \mathcal {G}^{3} \, \sum _{\ell \ge m}{\mathcal {G}_{\ell } x^{\ell }} + \mathcal {G}^{4} x^{-n} \Big ) \, {\left| \left| \left| Q \right| \right| \right| }_{m+n,x} . \end{aligned}$$

This immediately yields the last statement of the corollary. $\square$

#### Theorem 4.10

The map $L:\mathcal {A}_{\mathbb {N}} \longrightarrow \mathcal {A}_{\mathbb {N}}$ has a fixed point $h \in \mathcal {A}_{\mathbb {N}}$ satisfying:

(i) $h \in \mathcal {A}_{\mathbb {N}}(x)$ for every $x > 1$ such that $\sum _{\ell \ge 1}{\mathcal {G}_{\ell } \, x^{\ell } } < \infty$,
(ii) $$\mathcal {G}^{-4} \mathbb {1} \le h \le \mathcal {G}^{4} \, \mathbb {1}$$ and $$\nu (h) = 1$$.

#### Proof

Let us consider the set

$$\begin{aligned} \mathcal {C} := \overline{{\text {conv}}}{\{ L^{n}(\mathbb {1}) :n \in \mathbb {N} \}}, \end{aligned}$$

which is clearly convex, closed and invariant by *L*, i.e. $L(\mathcal {C}) \subset \mathcal {C}$. Using Corollary 4.9, we easily deduce that for every $Q \in \mathcal {C}$ and $\ell \ge 1$

$$\begin{aligned} \nu (Q) = 1,\quad \Vert Q\Vert \le \mathcal {G}^{4}, \quad \Vert Q\Vert _{\ell } \le 2 \mathcal {G}^{3} \, \mathcal {G}_{\ell }\quad \text{ and } \quad \mathcal {G}^{-4} \mathbb {1} \le Q \le \mathcal {G}^{4} \mathbb {1}. \end{aligned}$$

In particular, $\mathcal {C} \subset \mathcal {A}_{\mathbb {N}}(x)$ whenever $x>1$ satisfies $\sum _{\ell \ge 1}{\mathcal {G}_{\ell } \, x^{\ell }} < \infty$, the set $\mathcal {C}$ is bounded and satisfies

45 $$\begin{aligned} \mathcal C \subset \mathcal {A}_{[1,\ell ]} \, + \, (2 \, \mathcal {G}^{3} \, \mathcal {G}_{\ell }) \, \mathbb {B}_{\mathcal {A}_{\mathbb {N}}}, \end{aligned}$$

where $\mathbb {B}_{\mathcal {A}_{\mathbb {N}}}$ is the closed unit ball of $\mathcal {A}_{\mathbb {N}}$. Since $\mathcal {A}_{[1,\ell ]}$ is finite-dimensional, the set $\mathcal {C}$ is moreover totally bounded, and thus compact in the norm topology. Applying again Schauder–Tychonov theorem (see [42, Theorem 5.28]) to $L|_{\mathcal {C}}:\mathcal {C} \longrightarrow \mathcal {C}$ we deduce the existence of $h \in \mathcal {C}$ with $L(h) = h$. $\square$

### Convergence of $L^{n}$

#### Theorem 4.11

Let $x > 1$ with $\sum _{\ell }{\mathcal {G}_{\ell } \, x^{\ell }} < \infty$. Then, there exists an absolute constant $C_{x} > 1$ with the following property: for every $a>0$ there is $N = N(x,a)$ such that for every $n \ge N$ and every $Q \in \mathcal {A}_{\mathbb {N}}$ positive and invertible with ${\left| \left| \left| Q \right| \right| \right| }_{1,x} \Vert Q^{-1}\Vert \le a$, it holds that

$$\begin{aligned} {\left| \left| \left| L^{n}(Q) \right| \right| \right| }_{1,x} \, \Vert L^{n}(Q)^{-1}\Vert ^{-1} \, \le \, C_{x} . \end{aligned}$$

#### Proof

Let us fix $x>1$ as above. Given $a>0$, we are going to show that for every $3 \le r \in \mathbb {N}$, every $n \ge N:=3r$ and every $Q \in \mathcal {A}_{\mathbb {N}}$ positive and invertible with ${\left| \left| \left| Q \right| \right| \right| }_{1,x} \, \Vert Q^{-1}\Vert \le a$, it holds that

46 $$\begin{aligned} {\left| \left| \left| L^{n}(Q) \right| \right| \right| }_{1,x} \, \Vert L^{n}(Q)^{-1}\Vert \, \le K_{x}\, (\, 1 + a \, x^{-r} \,) \end{aligned}$$

for some constant $K_{x}$ independent of *a* and *r*. It is clear that the theorem follows from this statement by taking $r = r(a,x)$ large enough so that $a\le x^{r}$. To prove the statement, let us first write *n* in the form $n = 2r + r'$ with $r' \ge r$. On the one hand, we have using Theorem 4.4.(*iv*) and the fact that $L^{n} = \mathcal {L}^{n}/\mu ^{n}$

$$\begin{aligned} \Vert L^{2r+r'}(Q) \Vert \, \Vert L^{2r+r'}(Q)^{-1}\Vert \, \le \, \mathcal {G}^{4} \, \Vert L^{2r}(Q) \Vert \, \Vert L^{2r}(Q)^{-1}\Vert . \end{aligned}$$

On the other hand, for each $\ell \ge 1$ we can apply Theorem 4.4.(*v*) to obtain

$$\begin{aligned} \Vert L^{2r + r'}&(Q) \Vert _{\ell } \, \Vert L^{2r + r'}(Q)^{-1}\Vert = \Vert L^{r'} (L^{2r}(Q))\Vert _{\ell } \, \Vert L^{r'} (L^{2r} (Q))^{-1}\Vert \\&\le 2 \mathcal {G}^{3} \, \mathcal {G}_{\ell } \, \Vert L^{2r}(Q)\Vert \, \Vert L^{2r}(Q)^{-1}\Vert + \mathcal {G}^{4} \, \Vert L^{2r}(Q)\Vert _{r'+\ell } \, \Vert L^{2r}(Q)^{-1}\Vert . \end{aligned}$$

Combining the last two inequalities we get that

47 $$\begin{aligned} \begin{aligned} {\left| \left| \left| L^{n}(Q) \right| \right| \right| }_{1,x} \, \Vert L^{n} (Q)^{-1}\Vert \,&= \, \Vert L^{n}(Q) \Vert \, \Vert L^{n}(Q)^{-1}\Vert \\&\quad + \, \sum _{\ell \ge 1} \, \Vert L^{n}(Q) \Vert _{\ell } \, \Vert L^{n}(Q)^{-1}\Vert \, x^{\ell }\\&\, \le \, 2 \, \mathcal {G}^{4} \, \Big ( 1 + \sum _{\ell \ge 1} \mathcal {G}_{\ell } \, x^{\ell } \Big ) \, \Vert L^{2r}(Q)\Vert \, \Vert L^{2r}(Q)^{-1}\Vert \\&\quad + \mathcal {G}^{4} \, \sum _{\ell \ge 1}{\Vert L^{2r}(Q)\Vert _{r'+\ell } \, \Vert L^{2r}(Q)^{-1} \Vert \, x^{\ell }}.\\ \end{aligned} \end{aligned}$$

Let us focus on the last expression of (47). To bound the first summand we can apply Theorem 4.5 with $n= 2r$ and $\ell = r$, so that

48 $$\begin{aligned} \begin{aligned} \Vert L^{2r}(Q)\Vert \, \Vert L^{2r}(Q)^{-1}\Vert \,&\le \, \mathcal {G}^{4} \left( 1 + 4 \mathcal {G}^{3} \mathcal {G}_{r} \Vert Q\Vert \, \Vert Q^{-1}\Vert + 4 \mathcal {G}^{4} \, \Vert Q\Vert _{r} \, \Vert Q^{-1}\Vert \right) \\&\le \, \mathcal {G}^{4} \, \left( 1 + 4 \mathcal {G}^{3} \mathcal {G}_{r} a + 4 \mathcal {G}^{4} a x^{-r} \right) \\&\le \mathcal {G}^{4} \, (4 \mathcal {G}^{3} \, \mathcal {G}_{r} \,x^{r} +4 \mathcal {G}^{4} ) \, \left( 1 + a \, x^{-r} \right) . \end{aligned} \end{aligned}$$

A similar idea works for the second summand if we first apply Theorem 4.4.(v)

49 $$\begin{aligned} \begin{aligned}&\sum _{\ell \ge 1} \Vert L^{2r} (Q)\Vert _{r'+\ell } \, \Vert L^{2r}(Q)^{-1} \Vert \, x^{\ell } \\&\quad \le \, 2 \, \mathcal {G}^{3}\, \sum _{\ell \ge 1} \, \mathcal {G}_{r'+\ell } \, x^{\ell } \, \Vert Q\Vert \, \Vert Q^{-1}\Vert \, + \, \mathcal {G}^{4} \, \sum _{\ell \ge 1} \Vert Q\Vert _{2r+r'+\ell } \, \Vert Q^{-1}\Vert \, x^{\ell }\\&\quad \le \, 2 \, \mathcal {G}^{4} \, \Big ( 1 + \sum _{\ell \ge 1}{\mathcal {G}_{\ell } \, x^{\ell }}\Big ) \,a \, x^{-r}. \ \end{aligned} \end{aligned}$$

Finally, applying (48) and (49) to (47) we conclude that the statement (46) holds. $\square$

The previous result is the key ingredient for the following main theorem.

#### Theorem 4.12

Let $x>1$ with $\sum _{\ell \ge 1}{\mathcal {G}_{\ell } \, x^{\ell }} < \infty$. Then, there exist $K_{x}>0$ and $\delta _{x} > 0$ with the following property: for every $n \in \mathbb {N}$ and $Q \in \mathcal {A}_{\mathbb {N}}(x)$

$$\begin{aligned} {\left| \left| \left| L^{n}(Q) - \nu (Q) h \right| \right| \right| }_{1,x} \le K_{x} \, e^{- n \, \delta _{x}} \, {\left| \left| \left| Q \right| \right| \right| }_{1,x}. \end{aligned}$$

#### Proof

Let $C = C_{x}>1$ be the constant provided in Theorem 4.11 (that we can assume to be greater than three) and let $N = N(x)$ be the corresponding number when applying the aforementioned theorem with $a = 2 C$. This means that if $Q \in \mathcal {A}_{\mathbb {N}}$ is positive, invertible and satisfies

$$\begin{aligned} {\left| \left| \left| Q \right| \right| \right| }_{1,x} \, \Vert Q^{-1}\Vert \, \le \, 2 C, \end{aligned}$$

then for every $n \ge N$

$$\begin{aligned} {\left| \left| \left| L^{n}(Q) \right| \right| \right| }_{1,x} \, \le \, C \, \Vert L^{n}(Q)^{-1}\Vert ^{-1} , \end{aligned}$$

and so using that $\nu (L^{n}(Q)) = \nu (Q)$ we get

$$\begin{aligned} \nu (Q) \, \le \, {\left| \left| \left| L^{n}(Q) \right| \right| \right| }_{1,x} \, \le \, C \, \Vert L^{n}(Q)^{-1}\Vert ^{-1} \le C \, \nu (Q). \end{aligned}$$

This yields that the linear operator $\psi _{N}: \mathcal {A}_{\mathbb {N}} \longrightarrow \mathcal {A}_{\mathbb {N}}$ given by

$$\begin{aligned} \psi _{N}(A) = L^{N}(A) - \frac{\nu (A)}{2C} \, \mathbb {1}, \quad A \in \mathcal {A}_{\mathbb {N}} \end{aligned}$$

satisfies for the above *Q* that $\psi _{N}(Q)$ is positive and invertible with

$$\begin{aligned} \Vert \psi _{N}(Q) ^{-1} \Vert ^{-1} \ge \Vert L^{N}(Q)^{-1}\Vert ^{-1} - \frac{\nu (Q)}{2C} \, \ge \, \frac{\nu (Q)}{2C} \ge \frac{\Vert L^{N}(Q)^{-1}\Vert ^{-1}}{2} , \end{aligned}$$

and moreover for every $\ell \ge 1$

$$\begin{aligned} \Vert \psi _{N}(Q)\Vert \, \le \, \Vert L^{N}(Q)\Vert \quad \text{ and } \quad \Vert \psi _{N}(Q)\Vert _{\ell } \, = \, \Vert L^{N}(Q)\Vert _{\ell } . \end{aligned}$$

Altogether yields that the above *Q* satisfies

$$\begin{aligned} {\left| \left| \left| \psi _{N}(Q) \right| \right| \right| }_{1,x} \, \Vert \psi _{N}(Q)^{-1} \Vert \, \le \, 2 \, {\left| \left| \left| L^{N}(Q) \right| \right| \right| }_{1,x} \, \Vert L^{N}(Q)^{-1} \Vert \, \le \, 2 C. \end{aligned}$$

We can iterate this process and get that for every $k \in \mathbb {N}$

$$\begin{aligned} {\left| \left| \left| \psi _{N}^{k}(Q) \right| \right| \right| }_{1,x} \, \left\| \psi _{N}^{k}(Q)^{-1} \right\| \, \le \, 2 C. \end{aligned}$$

Consequently

50 $$\begin{aligned} {\left| \left| \left| \psi _{N}^{k}(Q) \right| \right| \right| }_{1,x} \, \le \, 2 C\, \left\| \psi _{N}^{k}(Q)^{-1} \right\| ^{-1} \, \le \, 2 C \nu ( \psi _{N}^{k}(Q) ) = 2 C \, \left( 1 - \frac{1}{2C} \right) ^{k} \nu (Q). \end{aligned}$$

Having this observation in mind we can now prove the result. We are going to distinguish several cases or steps:

(i) Let  be a self-adjoint element with $\nu (Q) = 0$ and . Write  and  which are both positive and invertible. Then

$$\begin{aligned} {{\left| \left| \left| Q_{i} \right| \right| \right| }_{1,x} \Vert Q_{i}^{-1}\Vert \, \le \, {\left| \left| \left| Q_{i} \right| \right| \right| }_{1,x} \, \le \, 2 + {\left| \left| \left| Q \right| \right| \right| }_{1,x} \, \le \, 3\, \le C \, \quad (i=1,2).} \end{aligned}$$

Thus we can apply (50) to get that for every $k \in {\mathbb {N}}$

$$\begin{aligned} {{\left| \left| \left| \psi _{N}^{k}(Q_{i}) \right| \right| \right| }_{1,x} \le 4 C \, \Big ( 1 - \frac{1}{2C}\Big )^{k} \quad (i=1,2).} \end{aligned}$$

But since $\nu (Q) = 0$, for every $k \in {\mathbb {N}}$

$$\begin{aligned} {{\left| \left| \left| L^{kN}\big ( Q \big ) \right| \right| \right| }_{1,x} \!=\!{\left| \left| \left| \psi _{N}^{k}(Q) \right| \right| \right| }_{1,x}\le {\left| \left| \left| \psi _{N}^{k}(Q_{1}) \right| \right| \right| }_{1,x}\!+\! {\left| \left| \left| \psi _{N}^{k}(Q_{2}) \right| \right| \right| }_{1,x} \le 8 C\Big ( 1 - \frac{1}{2C} \Big )^{k}.} \end{aligned}$$

If we take an arbitrary $n \in {\mathbb {N}}$ and write it as $n = k N + r$ with  and , then we deduce from the previous inequality and the second part of Corollary 4.9 that

$$\begin{aligned} {{\left| \left| \left| L^{n}(Q) \right| \right| \right| }_{1,x}\!=\!{\left| \left| \left| L^{r} \big ( L^{k N}(Q) \big ) \right| \right| \right| }_{1,x}\le 8 C \, {\left| \left| \left| L^{r} \right| \right| \right| }_{1,x}\Big ( 1 - \frac{1}{2C}\Big )^{k}\le 8\, \widetilde{C}_{x}\Big (1\!-\! \frac{1}{2C} \Big )^{\frac{n}{N\!+\!1}}.} \end{aligned}$$

Remark that the constants $\widetilde{C}_{x}, C, N$ only depend on *x*.

(ii) By linearity, we can extend the previous inequality to every $Q \in \mathcal {A}_{\mathbb {N}}(x)$ with $\nu (Q)=0$ (without restriction on the norm) as

$$\begin{aligned} {\left| \left| \left| L^{n}(Q) \right| \right| \right| }_{1,x} \, \le \, 8\, \widetilde{C}_{x} \, \Big ( 1 - \frac{1}{2C} \Big )^{\frac{n}{N+1}} {\left| \left| \left| Q \right| \right| \right| }_{1,x}. \end{aligned}$$

(iii) Let $Q \in \mathcal {A}_{\mathbb {N}}(x)$ be a self-adjoint element. We can apply to $Q' = Q - \nu (Q) h$ the estimate from (*ii*) that

$$\begin{aligned} {\left| \left| \left| L^{n}(Q) - \nu (Q) h \right| \right| \right| }_{1,x} = {\left| \left| \left| L^{n}(Q') \right| \right| \right| }_{1,x} \,&\le \, 8 \, \widetilde{C}_{x} \, \Big ( 1 - \frac{1}{2C} \Big )^{\frac{n}{N+1}}\ {\left| \left| \left| Q - \nu (Q) h \right| \right| \right| }_{1,x}\\&\le 8 \, \widetilde{C}_{x} \, (1 + {\left| \left| \left| h \right| \right| \right| }_{1,x}) \, \Big ( 1 - \frac{1}{2C} \Big )^{\frac{n}{N+1}}\ {\left| \left| \left| Q \right| \right| \right| }_{1,x}\, \end{aligned}$$

(iv) Finally, for an arbitrary $Q \in \mathcal {A}_{\mathbb {N}}(x)$, we can use the usual decomposition $Q=Q_{1} + iQ_{2}$ where $Q_{1} = (Q+Q^{\dagger })/2$ and $Q_{2} = (iQ^{\dagger } - i Q)/2$ are self-adjoint, and moreover ${\left| \left| \left| Q_{i} \right| \right| \right| }_{1,x} \le {\left| \left| \left| Q \right| \right| \right| }_{1,x}$ ($i=1,2$). Applying the estimate from (*iii*) to each $Q_{i}$ we easily conclude the result. $\square$

#### Corollary 4.13

Let $x>1$ with $\sum _{\ell \ge 1}{\mathcal {G}_{\ell } \, x^{\ell }} < \infty$. Then, there exist $\widetilde{K}_{x}>0$ and $\delta _{x} > 0$ with the following property: for every $m, n \ge 0$ and $Q \in \mathcal {A}_{\mathbb {N}}$ it holds that

$$\begin{aligned} {\left| \left| \left| L^{n+m}(Q) - \nu (Q) h \right| \right| \right| }_{1,x} \le \widetilde{K}_{x} \, e^{- n \, \delta _{x}} \, {\left| \left| \left| Q \right| \right| \right| }_{1+m,x}. \end{aligned}$$

In particular, if $Q \in \mathcal {A}_{[1,m]}$ then

$$\begin{aligned} {\left| \left| \left| L^{n+m}(Q) - \nu (Q) h \right| \right| \right| }_{1,x} \le \widetilde{K}_{x} \, e^{- n \, \delta _{x}} \, \Vert Q \Vert . \end{aligned}$$

#### Proof

Combining Theorem 4.12 and Corollary 4.9 we deduce that

$$\begin{aligned} {\left| \left| \left| L^{n+m}(Q) - \nu (Q) h \right| \right| \right| }_{1,x}&\, = \, {\left| \left| \left| L^{n}(L^{m}(Q)) - \nu (L^{m}(Q)) h \right| \right| \right| }_{1,x}\\&\, \le \, K_{x} \, e^{- n \, \delta _{x}} \, {\left| \left| \left| L^{m}(Q) \right| \right| \right| }_{1,x}\\&\, \le \, \widetilde{K}_{x} \, e^{- n \, \delta _{x}} \, {\left| \left| \left| Q \right| \right| \right| }_{m+1,x}. \end{aligned}$$

$$\square $$

### Exponential decay of correlations and convergence of states

#### Theorem 4.14

Let $n, \ell \ge 1$, $A \in \mathcal {A}_{[1,n]}$ and $B \in \mathcal {A}_{\mathbb {N}}$. Then,

51 $$\begin{aligned} \Vert L^{n}(A \, \tau _{n+\ell }(B)) - \tau _{\ell }(B) \, L^{n}(A) \Vert \le 4 \mathcal {G}^{3}\, \mathcal {G}_{\ell } \, \Vert A\Vert \, \Vert B\Vert . \end{aligned}$$

In particular, there exist constants $K, \delta > 0$ independent of $n, \ell$ and of the observables *A*, *B* such that

52 $$\begin{aligned} |\nu (A \, \tau _{n+\ell }(B)) - \nu (A) \, \nu (\tau _{n+\ell }(B))|\, \le \, K \, \Vert A \Vert \, \Vert B\Vert \, e^{- \delta \, \ell }. \end{aligned}$$

#### Proof

Let us fist notice that

53 $$\begin{aligned} \mathcal {L}_{(n,n+\ell )} \big (\, A \, \tau _{n+\ell }(B) \big ) \, = \, \tau _{\ell }(B) \, \mathcal {L}_{(n,n+\ell )}(A), \end{aligned}$$

since the support of $\tau _{n+\ell }(B)$ is contained in $[1+n+\ell , \infty )$ and so it is disjoint from the other factors and the lattice sites where the partial trace acts. We can then estimate, using Theorem 4.4

$$\begin{aligned} \Vert \mathcal {L}_{n}(A\tau _{n+\ell }(B)) - \mathcal {L}_{(n,n+\ell )}(A\tau _{n+\ell }(B))\Vert \,&\le \, {\textrm{tr}}\big ( e^{- H_{[1,n]}}\big )\, 2 \, \mathcal {G} \, \mathcal {G}_{\ell } \, \Vert A \, \tau _{n+\ell }(B)\Vert ,\\ \Vert \tau _{\ell }(B) \mathcal {L}_{n}(A) - \tau _{\ell }(B) \mathcal {L}_{(n,n+\ell )}(A)\Vert \,&\le \, {\textrm{tr}}\big ( e^{- H_{[1,n]}}\big )\, 2 \, \mathcal {G} \, \mathcal {G}_{\ell } \, \Vert A \Vert \, \Vert \tau _{\ell }(B)\Vert . \end{aligned}$$

Combining these inequalities with (53) we deduce

$$\begin{aligned} \Vert \mathcal {L}_{n}(A\tau _{n+\ell }(B)) - \tau _{\ell }(B) \mathcal {L}_{n}(A) \Vert \, \le \, {\textrm{tr}}(e^{-H_{[1,n]}}) \, 4 \mathcal {G} \, \mathcal {G}_{\ell } \, \Vert A\Vert \, \Vert B\Vert . \end{aligned}$$

Dividing by $\mu ^{n}$ on both sides and using (43) we conclude that (51) hold. Let us focus now on the second statement. We can assume w.l.o.g. that $\nu (A) = 0$ replacing *A* with $A - \nu (A) \mathbb {1}$. Let $r := \lfloor \ell /2\rfloor$, then

$$\begin{aligned} |\nu (A \tau _{n+\ell }(B))|&= |\nu (L^{n+r}(A\tau _{n+\ell }(B)))| \le \Vert L^{n+r}(A\tau _{n+\ell }(B))\Vert \\&\le \, \Vert L^{n+r}(A\tau _{n+\ell }(B)) - \tau _{\ell }(B) \, L^{n+r}(A)\Vert + \Vert B\Vert \, \Vert L^{n+r}(A)\Vert . \end{aligned}$$

Applying (51) and Corollary 4.13, we have that for suitable constants $\widetilde{K} ,\widetilde{\delta } > 0$ independent of *n*, *r*

$$\begin{aligned} |\nu (A \tau _{n+\ell }(B))| \le 2 \, \mathcal {G}^{3} \, \mathcal {G}_{\ell -r} \, \Vert A\Vert \, \Vert B\Vert + \widetilde{K} \, \Vert A \Vert \, \Vert B\Vert \, e^{-r \, \widetilde{\delta }} \end{aligned}$$

where $\mathcal {G}_{\ell - r} \le \mathcal {G}_{r}$ since $\ell - r \ge r$, so using that $\mathcal {G}_{r}$ converges to zero exponentially fast in *r* we conclude the result. $\square$

#### Lemma 4.15

Let $x>1$ with $\sum _{\ell } \mathcal {G}_{\ell } \, x^{\ell } < \infty$. Then, there are constants $K_{x}, \delta _{x}>0$ such that for every $a,k \ge 1$ and every $Q \in \mathcal {A}_{\mathbb {N}}(x)$

$$\begin{aligned} \left| \varphi ^{[1,a+k]}(Q) - \nu (Q) \right| \, \le \, K_{x} \, e^{- \delta _{x} a} \, {\left| \left| \left| Q \right| \right| \right| }_{1 + k, x}. \end{aligned}$$

In particular, the sequence of states $\varphi ^{[1,k]}$ converges pointwise on $\mathcal {A}_{\mathbb {N}}$ to the state $\nu$.

#### Proof

We can write for every $1 \le n < a':=a+k$

$$\begin{aligned} \varphi ^{[1,a']}(Q) \, = \, \frac{\varphi ^{[1,a'-n]}\left( L_{(n,a')}(Q) \right) }{\varphi ^{[1,a'-n]}\left( L_{(n,a')}(\mathbb {1})\right) }, \quad \nu (Q) = \frac{\varphi ^{[1,a'-n]}\left( \nu (Q) h\right) }{\varphi ^{[1,a'-n]}\left( h\right) } \end{aligned}$$

where the first identity already appeared in (34). Thus

$$\begin{aligned} \left| \varphi ^{[1,a']}(Q) - \nu (Q) \right| \, \le \, \big | \varphi ^{[1,a']}(Q)\big | \, \frac{ \Vert L_{(n,a')}(\mathbb {1}) - h \Vert }{\varphi ^{[1,a'-n]}(h)} \, + \frac{\Vert L_{(n,a')}(Q) - \nu (Q) h \Vert }{\varphi ^{[1,a'-n]}(h)}. \end{aligned}$$

By Corollary 4.9.(*ii*) and Theorem 4.12

$$\begin{aligned} \Vert L_{(n,a')}(Q) - \nu (Q)h \Vert&\le \Vert L_{(n,a')}(Q) - L^{n}(Q)\Vert + \Vert L^{n}(Q) - \nu (Q) h\Vert \\&\le 2 \, \mathcal {G}^{3} \, \mathcal {G}_{a'-n} \, \Vert Q\Vert + \widetilde{K}_{x} \, e^{-(n-k) \tilde{\delta }_{x}} {\left| \left| \left| Q \right| \right| \right| }_{1+k, x}. \end{aligned}$$

Moreover, applying the state $\varphi ^{[1,a'-n]}$ to Theorem 4.10.(*ii*) we get $\varphi ^{[1,a'-n]}(h) \ge \mathcal {G}^{-2}$. Therefore, combining these estimates

$$\begin{aligned} \left| \varphi ^{[1,a']}(Q) - \nu (Q) \right| \, \le \, 4 \, \mathcal {G}^{5} \, \mathcal {G}_{a'-n} \, \Vert Q\Vert + \mathcal {G}^{2} \, \widetilde{K}_{x} \, e^{-(n-k) \, \tilde{\delta }_{x}} \, {\left| \left| \left| Q \right| \right| \right| }_{1+k, x}. \end{aligned}$$

Finally, if we take $n := \lfloor a/2\rfloor + k$, we have that $n-k = \lfloor a/2\rfloor$ and $a'-n \ge \lfloor a/2\rfloor$, so that $\mathcal {G}_{a'-n} \le \mathcal {G}_{\lfloor a/2 \rfloor }$. Since the latter converges to zero exponentially fast in *a*, we conclude the result. $\square$

#### Theorem 4.16

There exist a translation invariant state $\psi$ on $\mathcal {A}_{\mathbb {N}}$ and $\delta >0$ such that

54 $$\begin{aligned} \lim _{k \rightarrow \infty } \, \Vert \nu \circ \tau _{k} - \psi \Vert \, e^{\delta k} \, = \, 0 . \end{aligned}$$

Moreover, the (unique) extension of $\psi$ to a translation invariant state on $\mathcal {A}_{\mathbb {Z}}$, that we also denote by $\psi$, satisfies that there are constants $K, \delta >0$ such that:

(i) For every $j \in \mathbb {Z}, \, k \in \mathbb {N}$ and every $A \in \mathcal {A}_{(-\infty , j]}$ and $B \in \mathcal {A}_{[j+k, \infty )}$
  $$\begin{aligned} |\psi (AB) - \psi (A) \, \psi (B)| \, \le \, \Vert A\Vert \, \Vert B\Vert \, K \, e^{ - \delta k}. \end{aligned}$$
(ii) For every $Q \in \mathcal {A}_{[a,b]}$ and every $k \in \mathbb {N}$
  $$\begin{aligned} \left| \psi (Q) - \varphi ^{[a-k,b+k]}(Q) \right| \le K e^{- \delta k} . \end{aligned}$$

In other words, $\psi$ is the infinite-volume KMS state associated to $\Phi$ at temperature one and satisfies exponential decay of correlations.

#### Proof

Let $n \in \mathbb {N}$, $N \ge 2$ for which we denote $N':=\lfloor N/2 \rfloor$, and $Q \in \mathcal {A}_{\mathbb {N}}$. Using Theorems 4.14 and 4.12 we have

$$\begin{aligned} \left| \nu \left( \tau _{n+N}(Q) \right) - \nu \left( \tau _{N}(Q) h\right) \right| \,&= \, \left| \nu \big (L^{n+N'}\big ( \tau _{N +n}(Q)\big ) \big ) - \nu \big (\tau _{N}(Q)h\big ) \right| \\&\quad \le \Vert L^{n+N'}\big ( \tau _{N+n}(Q) \big ) - \tau _{N}(Q) \, L^{n+N'}(\mathbb {1}) \Vert \\&\qquad + \, \Vert Q\Vert \, \Vert L^{n+N'}(\mathbb {1}) - h\Vert \\&\quad \le \, \big ( 4 \mathcal {G}^{3} \mathcal {G}_{N-N'} \, + \, K_{x} \, e^{- N' \, \delta _{x}} \big ) \, \Vert Q\Vert . \end{aligned}$$

As a consequence, $(\nu \circ \tau _{k})_{k}$ is a Cauchy sequence of states on $\mathcal {A}_{\mathbb {N}}$ convergent to a state $\psi$ over $\mathcal {A}_{\mathbb {N}}$ satisfying moreover

$$\begin{aligned} \Vert \nu \circ \tau _{N} - \psi \Vert \, \le \, 8 \, \mathcal {G} \, \mathcal {G}_{N-N'} + 2 K_{x} e^{- N' \, \delta _{x}}. \end{aligned}$$

Since the right hand-side of the previous expression converges to zero exponentially fast with *N*, we deduce that (54) holds for some $\delta >0$. Moreover, $\psi$ is translation invariant since $\psi \circ \tau = \lim _{k} \nu \circ \tau _{k} \circ \tau = \lim _{k} \nu \circ \tau _{k+1} = \psi$, and so it can be uniquely extended to a translationally invariant state over $\mathcal {A}$ also denoted by $\psi$. Let us now check statements (i) and (ii).

(i) Let $A \in \mathcal {A}_{(-\infty , j]}$ and $B \in \mathcal {A}_{[j+k, \infty )}$ as above. We can assume w.l.o.g. that *A* is a local observable. Then, there exist $n_{0} \in \mathbb {N}$ such that $\tau _{n}(A), \tau _{n}(B) \in \mathcal {A}_{\mathbb {N}}$ for every $n \ge n_{0}$. We have by Theorem 4.14 that there are constants $K, \delta > 0$ independent of $k,j,n_{0},n$ such that

$$\begin{aligned} |\nu (\tau _{n}(A) \tau _{n}(B)) - \nu (\tau _{n}(A)) \nu (\tau _{n}(B))| \le K e^{- \delta k} \,\Vert A\Vert \, \Vert B\Vert . \end{aligned}$$

Taking limit on *n* and using (54) we conclude the result.

(ii) Let $Q \in \mathcal {A}_{[a,b]}$ and $k \in \mathbb {N}$. Then

$$\begin{aligned} \left| \varphi ^{[a-k,b+k]}(Q) - \psi (Q) \right| \,&=\, \left| \varphi ^{[1,1+b-a+2k]}(\tau _{1+k-a}(Q)) - \psi (Q)\right| \\&\le \, \left| \varphi ^{[1,1+b-a+2k]}(\tau _{1+k-a}(Q)) - \nu (\tau _{1+k-a}(Q))\right| \\&\quad + \left| \nu (\tau _{1+k-a}(Q)) - \psi (Q)\right| . \end{aligned}$$

The first summand of the last expression can be estimated using first Lemma 4.15

$$\begin{aligned} \left| \varphi ^{[1,1+b-a+2k]}(\tau _{1+k-a}(Q)) - \nu (\tau _{1+k-a}(Q))\right| \le K_{x} \, e^{-(k-1) \, \delta _{x}} {\left| \left| \left| \tau _{1+k-a}(Q) \right| \right| \right| }_{x, 2+b-a+k}, \end{aligned}$$

and then using that $\tau _{1+k-a}(Q)$ has support in $[1+k,1+b-a+k]$, so that

$$\begin{aligned} {\left| \left| \left| \tau _{1+k-a}(Q) \right| \right| \right| }_{x, 2+b-a+k} = \Vert \tau _{1+k-a}(Q) \Vert = \Vert Q\Vert . \end{aligned}$$

The second summand can be estimated replacing $\psi (Q) = \psi (\tau _{1-a}(Q))$ by translational invariance and using (54). $\square$

## Spectral Gap Problem for 2D PEPS

In this section, we apply the locality estimates from Sect. 2 to extend a result from [22] to interactions with exponential decay. To explain the result, we first need to recall some concepts from the theory of *Projected Entangled Pair States* (PEPS).

### Basics

Although PEPS can be defined on general graphs and lattices, we are going to restrict for simplicity to the two-dimensional lattice $\mathbb {Z}^{2}$, and denote by $\mathcal {E}$ its set of edges. Fixed $d, D \in \mathbb {N}$, we consider at each lattice point $v \in \mathbb {Z}^{2}$ a linear operator or tensor
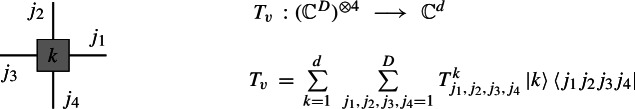


Here, $\mathcal {H}_{v} \equiv \mathbb {C}^{d}$ is the *physical* space associated to *v* and each $\mathbb {C}^{D}$ is the *virtual* space corresponding to an edge *e* incident to *v*. We will refer to this family of operators $(T_{v})_{v}$ as a PEPS. For each finite region $\mathcal {R} \subset \mathbb {Z}^{2}$ let us denote by $\mathcal {E}_{\mathcal {R}}$ the set of edges connecting vertices in $\mathcal {R}$, and by $\mathcal {E}_{\partial \mathcal {R}}$ the edges simultaneously incident to $\mathcal {R}$ and $\mathbb {Z}^{2} {\setminus } \mathcal {R}$. We can then associate to $\mathcal {R}$ the linear operator

$$\begin{aligned} \bigotimes _{v \in \mathcal {R}}T_{v} \,: \, \bigotimes _{e \in \mathcal {E}_{\mathcal {R}}} \left( \mathbb {C}^{D} \otimes \mathbb {C}^{D}\right) \otimes \bigotimes _{e \in \mathcal {E}_{\partial \mathcal {R}}} \mathbb {C}^{D}\,\, \longrightarrow \,\, \bigotimes _{v \in \mathcal {R}}\mathbb {C}^{d}. \end{aligned}$$

**Fig. 2:**
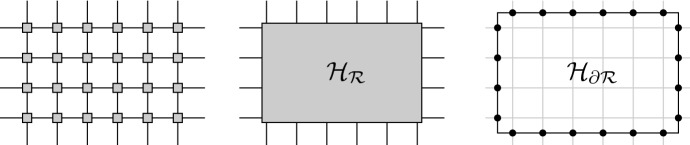
Contracted tensors on a finite rectangular region $\mathcal {R}$

If we set on each edge $e \in \mathcal {E}_{\mathcal {R}}$ a maximally entangled statethen, we get a linear operator (see Fig. 2) from the virtual space $\mathcal {H}_{\partial \mathcal {R}} := \bigotimes _{e \in \mathcal {E}_{\partial \mathcal {R}}}{\mathbb {C}^{D}}$ into the bulk physical space $\mathcal {H}_{\mathcal {R}} := \bigotimes _{v \in \mathcal {R}}{\mathbb {C}^{d}}$We say that the PEPS is *injective* on $\mathcal {R}$ if the map $T_{\mathcal {R}}$ is injective. This property is somehow generic on regions large enough, and allows to construct a local Hamiltonian for which the PEPS is its unique ground state. Indeed, let us assume that every $T_{v}$ is injective. For each edge $e=\{v_{1}, v_{2}\}$ of the lattice, let $h_{e}$ denote the orthogonal projection onto the orthogonal complement of ${\text {Im}} T_{\{ v_{1},v_{2}\}}$. Then, the set of nearest-neighbor interactions $(h_{e})_{e \in \mathcal {E}}$ determines the *parent Hamiltonian* of the PEPS. It satisfies that for every finite region $\mathcal {R}$ of the lattice, the groundspace of $H_{\mathcal {R}} = \sum _{e \in \mathcal {E}_{\mathcal {R}}}{h_{e}}$ coincides with ${\text {Im}}T_{\mathcal {R}}$. Moreover, it is *frustration-free*, namely that every ground state of $H_{\mathcal {R}}$ is a ground state of every local interaction term. See [40] for a detailed exposition of these statements.

Finally, let us recall that the *boundary state* of the PEPS on $\mathcal {R}$ is defined as^1^

$$\begin{aligned} \rho _{\partial \mathcal {R}} := T_{\mathcal {R}}^{\dagger } T_{\mathcal {R}}\, \in \, \mathcal {B}(\mathcal {H}_{\partial \mathcal {R}}) . \end{aligned}$$

Under the injectivity condition the boundary state is a full-rank positive matrix, and so it can be written as

$$\begin{aligned} \rho _{\partial \mathcal {R}} = \exp (2 \,G_{\partial \mathcal {R}}) \end{aligned}$$

for some self-adjoint operator $G_{ \partial \mathcal {R}} \in \mathcal {B}(\mathcal {H}_{\partial \mathcal {R}})$ called the *boundary Hamiltonian*. The choice of a factor two in the exponent is just convenient for later arguments.

### Spectral gap and approximate factorization

A main problem to tackle is finding conditions ensuring that the family of parent Hamiltonians $(H_{\mathcal {R}})_{\mathcal {R}}$ where $\mathcal {R}$ runs over all (or a certain family of) finite rectangles is *gapped*, namely that

$$\begin{aligned} \inf _{\mathcal {R}}{\gamma (H_\mathcal {R})} > 0, \end{aligned}$$

where $\gamma (H_{\mathcal {R}})$ is the difference between the two smallest eigenvalues of $H_{\mathcal {R}}$. This issue is related to the correlation properties in the bulk of the system, and the latter are connected to the locality features of the boundary states and Hamiltonians [9]. This relation was formalized in [22] as a sufficient condition on the boundary states for the parent Hamiltonian to be gapped. We have to introduce some notation prior to the formal statement.

#### Definition 5.1

**Fig. 3:**
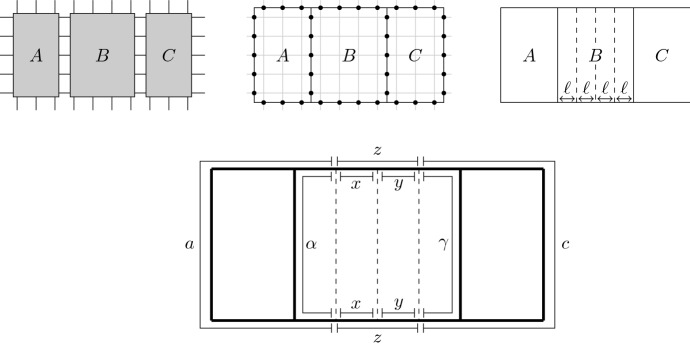
*A*, *B*, *C* are $\ell$-admissible rectangles. At the bottom, notation introduced for boundary segments

Given a length parameter $\ell \in \mathbb {N}$, let us say that three given rectangles *A*, *B*, *C* are $\ell$-*admissible* if they are adjacent, *B* shields *A* from *C*, and the width of *B* is at least $4\ell$, as portrayed in Fig. 3.

In this setup, let us introduce in Fig. 3 some further notation for the boundary regions of *ABC* that we will use in the following subsections. The horizontal sides of *B* are split into four segments of length greater than the length scale $\ell$. Notice that *z* and so *x*, *y* are at distance greater than $\ell$ from $\partial A \cup \partial C$, while *a* and $\alpha$ (as well as *c* and $\gamma$) overlap on segments with length greater than $\ell$, by definition.

#### Definition 5.2

Let us say that a family of positive and invertible observables $(\rho _{\partial \mathcal {R}})_{\mathcal {R}}$, where $\mathcal {R}$ runs over all finite rectangles, is *approximately factorizable* if there is a positive decreasing function $\ell \longmapsto \varepsilon (\ell )$ with polynomial decay, i.e. $\varepsilon (\ell ) = {\text {poly}}(1/\ell )$, fulfilling that for every $\ell$-admissible rectangles *ABC* there exist invertible observables $\Delta _{az}, \Delta _{zc}, \Upsilon _{\alpha z}, \Upsilon _{z \gamma }$ on $\partial A \cup \partial B \cup \partial C$ (subscripts indicate the support according to Fig. 3) such that the full-rank matrices

$$\begin{aligned} \sigma _{\partial ABC}:= \Delta _{zc} \Delta _{az}, \,\,\, \sigma _{\partial AB}:=\Upsilon _{z \gamma } \Delta _{az},\,\,\, \sigma _{\partial BC}:=\Delta _{zc} \Upsilon _{\alpha z}, \,\,\, \sigma _{\partial B} = \Upsilon _{z \gamma } \Upsilon _{\alpha z}\, \end{aligned}$$

satisfy

$$\begin{aligned} \Vert \rho _{\partial \mathcal {R}}^{1/2} \, \sigma _{\partial \mathcal {R}}^{-1} \, \rho _{\partial \mathcal {R}}^{1/2} \, - \, \mathbb {1}\Vert < \varepsilon (\ell )\quad \text{ for } \text{ every } \mathcal {R} \in \{ABC, AB, BC, B \}. \end{aligned}$$

If the boundary states of the above injective PEPS satisfy this property, then the family of parent Hamiltonians $(H_{\mathcal {R}})_{\mathcal {R}}$ where $\mathcal {R}$ runs over all finite rectangles in $\mathbb {Z}^{2}$ is gapped [22]. Moreover, the authors show that this approximate factorization property is satisfied if the boundary Hamiltonians posses nice locality and compatibility conditions.

#### Definition 5.3

*(Locality and homogeneity).* A local decomposition of the boundary Hamiltonians $(G_{\partial \mathcal {R}})_{\mathcal {R}}$ is simply a decomposition of each term as a sum of local interactions

$$\begin{aligned} G_{\partial \mathcal {R}}= \sum _{X \subset \partial \mathcal {R}}{g_{X}^{\partial \mathcal {R}}}, \end{aligned}$$

where each $g_{X}^{\partial \mathcal {R}}$ is supported in the virtual indices corresponding to *X*. The rate of decay of this decomposition is defined as the sequence

$$\begin{aligned} \Omega _{k} := \, \sup _{\mathcal {R}} \,\,\, \sup _{x \in \partial \mathcal {R}} \,\, \sum _{\begin{array}{c} X \ni x \,\, \text {s.t.}\\ {\text {diam}}_{\mathcal {R}}(X) \ge k \end{array}} \Vert g_{X}^{\partial \mathcal {R}}\Vert \,, \,\, k \ge 0 \end{aligned}$$

where the diameter ${\text {diam}}_{\mathcal {R}}(\cdot )$ is calculated in terms of the intrinsic distance ${\text {dist}}_{\partial \mathcal {R}}$ of $\partial \mathcal {R}$ considered as a one-dimensional periodic lattice $\mathbb {Z}_{n}$. We will say that this local decomposition has exponential decay if there is $\lambda > 0$ such that

$$\begin{aligned} \Vert \Omega \Vert _{\lambda }:= \sum _{k=0}^{\infty } e^{\lambda k} \Omega _{k} < \infty . \end{aligned}$$

In addition, given a positive sequence $\ell \longmapsto \eta (\ell )$ converging to zero as $\ell$ tends to infinity, we will say that this decomposition is $\eta$-*homogeneous* if for every pair of adjacent rectangles *AB*, the corresponding boundary Hamiltonians $G_{\partial AB}$ and $G_{\partial A}$ satisfy that for each $X \subset \partial {A} {\setminus } \partial {B}$


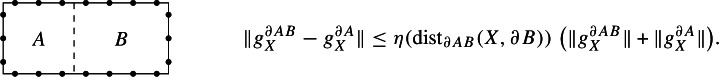


It is shown in [22, Section 5] that if the boundary Hamiltonians are finite-range, i.e. have a local decomposition such that $\Omega _{k}=0$ if *k* is larger than a fixed $r>0$ and the homogeneity condition holds for a controlling sequence $\eta$ that decays sufficiently (polynomially) fast, then the quasi-factorization condition holds. We aim to extend this result to interactions with exponential decay. Our method is analogous, based on the imaginary time locality estimate from previous sections, although slightly simpler in the sense that we replace the use of expansional formulas for multiple products with just perturbation formulas.

### Locality estimates on the boundary

First we have to obtain some locality estimates on the imaginary-time evolution of an observable $Q \in \mathcal {B}(\mathcal {H}_{\partial \mathcal {R}})$ with respect to a boundary Hamiltonian $G_{\partial \mathcal {R}}$. We are going to call a supporting set $\Lambda _{0} \subset \partial \mathcal {R}$ *admissible* if it consists of at most two connected segments. As usual, we denote

$$\begin{aligned} \Lambda _{k}:= \{ x \in \partial {\mathcal {R}} :{\text {dist}}_{\partial \mathcal {R}}(x, \Lambda _{0}) \le k \}\quad , \quad k \ge 0. \end{aligned}$$

#### Lemma 5.4

Let us consider a family of local boundary Hamiltonians $(G_{\partial \mathcal {R}})_\mathcal {R}$ with exponential decay $\Vert \Omega \Vert _{\lambda } < \infty$ for some $\lambda > 0$. Then, for every rectangle $\mathcal {R}$ and every observable $Q \in \mathcal {B}(\mathcal {H}_{\partial \mathcal {R}})$ with admissible support $\Lambda _{0}$ we have

55 $$\begin{aligned} \Vert \Gamma _{\Lambda _{L}}^{s}(Q) - \Gamma _{\Lambda _{\ell }}^{s}(Q) \Vert&\le \Vert Q \Vert e^{2 |s| \Omega _{0} |\Lambda _{0}| \, } \, e^{ 8 |s| \, \Vert \Omega \Vert _{\lambda } } \, e^{(8|s| \Omega _{0}- \lambda ) \ell }, \end{aligned}$$

56 $$\begin{aligned} \Vert \Gamma _{\Lambda _{L}}^{s}(Q)\Vert&\le \Vert Q \Vert e^{2 |s| \Omega _{0} |\Lambda _{0}| \, } \, e^{ 8 |s| \, \Vert \Omega \Vert _{\lambda } }, \end{aligned}$$

whenever $0 \le \ell \le L$ and $s \in \mathbb {C}$ satisfies $|s| \le \lambda /(8\Omega _{0})$.

#### Proof

Since $\Lambda _{0}$ consists of at most two connected segments, $|\Lambda _{k} {\setminus } \Lambda _{k-1}| \le 4$ for every $k \ge 1$. Consequently, and following the notation of Theorem 2.2, we can bound for every $0 \le j < k$

$$\begin{aligned} W(j, k) \,\, \le \,\, |\Lambda _{k} {\setminus } \Lambda _{k-1}| \, \Omega _{k-j} \,\, \le \,\, 4 \, \Omega _{k-j}. \end{aligned}$$

Analogously to Theorem 2.3, for every $0 \le \ell \le L$ and $s \in \mathbb {C}$

$$\begin{aligned} \Vert \Gamma _{\Lambda _{L}}^{s}(Q) - \Gamma _{\Lambda _{\ell }}^{s}(Q) \Vert&\le \Vert Q \Vert e^{2 |s| \Omega _{0} |\Lambda _{0}| \, } \, \sum _{k=\ell +1}^{L} e^{8 |s| \Omega _{0} k} \Omega _{k}^{*}(8 |s|),\\ \Vert \Gamma _{\Lambda _{L}}^{s}(Q)\Vert&\le \Vert Q \Vert e^{2 |s| \Omega _{0} |\Lambda _{0}| \, } \, \sum _{k=0}^{L} e^{8 |s| \Omega _{0} k} \Omega _{k}^{*}(8 |s|). \end{aligned}$$

Finally, we can argue as in the proof of (21)–(22) to obtain (55)–(56). $\square$

### Perturbation formulas on the boundary

**Fig. 4:**
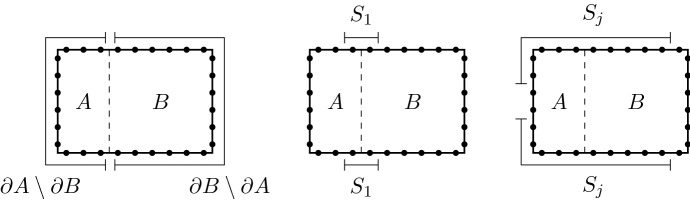
Adjacent rectangles *AB*

Let us consider the border of a rectangle *AB* made with two adjacent rectangles *A* and *B*. It is then formed by two connected segments, namely $\partial A {\setminus } \partial B$ and $\partial B {\setminus } \partial A$, see Fig. 4. Let us denote by $S_{1}$ the set containing the (four) extreme points of both segments, and

$$\begin{aligned} S_{j}:=\{ v \in \partial \mathcal {R} :{\text {dist}}_{\partial \mathcal {R}}(v, S_{1}) \le j \} \quad \text {for each}\quad j > 1. \end{aligned}$$

We are going to consider two local Hamiltonians on $\partial AB$, namely *G* and a perturbed version $\widetilde{G}=G+U$, and study bounds and locality properties of

57 $$\begin{aligned} e^{G}e^{-\widetilde{G}} = \mathbb {1} + \sum _{m=1}^{\infty } (-1)^{m} \int _{0}^{1}d \beta _{1} \cdots \int _{0}^{\beta _{m-1}}d \beta _{m} \, \Gamma _{G}^{-i \beta _{1}}(U) \cdots \Gamma _{G}^{-i \beta _{m}}(U), \end{aligned}$$

where we have used the expansional formulas described in Sect. 3.

#### Theorem 5.5

Let us assume that the boundary Hamiltonians $(G_{\partial \mathcal {R}})_{\mathcal {R}}$ have a local decomposition $(g_{\partial \mathcal R}^{X})$ with exponential decay $\Vert \Omega \Vert _{\lambda } < \infty$ for some $\lambda > 8 \Omega _{0}$. Then, for every pair of adjacent rectangles *AB* as above it holds that the local Hamiltonians

$$\begin{aligned} G := \sum _{X \subset \partial AB}{g_{X}^{\partial AB}}\quad \text{ and } \quad \widetilde{G} := \sum _{X \subset \partial A {\setminus } \partial B}{g_{X}^{\partial AB}} + \sum _{X \subset \partial B {\setminus } \partial A}{g_{X}^{\partial AB}} \end{aligned}$$

satisfy

$$\begin{aligned} \Vert e^{G}e^{-\widetilde{G}}\Vert&\le \exp { (e^{12 \Vert \Omega \Vert _{\lambda }}) }, \\ \Vert e^{G}e^{-\widetilde{G}} - e^{G_{S_{k}}} e^{\widetilde{G}_{S_{k}}}\Vert&\le \, \exp {(e^{12 \Vert \Omega \Vert _{\lambda }})} \, e^{12 \Vert \Omega \Vert _{\lambda }} \, e^{(8 \Omega _{0} - \lambda )k} . \end{aligned}$$

#### Proof

Let us denote by $\mathcal {F}$ the family of subsets *X* of $\partial AB$ such that $X \cap \partial A$ and $X \cap \partial B$ are both not empty. Then,

$$\begin{aligned} \widetilde{G} = G + U \quad \text{ where } \quad U = \sum _{X \in \mathcal {F}}{-g_{X}^{\partial AB}}. \end{aligned}$$

Next we split the family $\mathcal {F}$ into the following subfamilies: for every $j \ge 1$

$$\begin{aligned} \mathcal {F}_{j}:= \{ X \in \mathcal {F} :X \subset S_{j} \} {\setminus } \bigcup _{j' < j} {\mathcal {F}_{j'}}. \end{aligned}$$

We can then decompose

58 $$\begin{aligned} U = \sum _{j \ge 1}{U_{j}} \quad \text {and} \quad U_{S_{k}} = \sum _{j=1}^{k}{U_{j}}\,, \quad \text{ where } \quad U_{j}:=\sum _{X \in \mathcal {F}_{j}}{-g_{X}^{\partial AB}}. \end{aligned}$$

Since every set *X* in $\mathcal {F}_{j}$ has diameter greater than or equal to *j*, and it has non-empty intersection with $S_{j} {\setminus } S_{j-1}$ (that contains at most four points) we can easily deduce

$$\begin{aligned} \Vert U_{j}\Vert \le 4 \Omega _{j}. \end{aligned}$$

Moreover $U_{j}$ has support in $S_{j}$, which consists of at most two connected segments and contains at most 4*j* elements. This is an admissible supporting set, and so we can use the locality estimates from Lemma 5.4 to get for $|s| \le 1$ and every $j\le k$

$$\begin{aligned} \Vert \Gamma _{G}^{s}(U_{j}) \Vert , \Vert \Gamma _{G_{S_{k}}}^{s}(U_{j}) \Vert \,&\le \, \Vert U_{j}\Vert \, e^{2 \Omega _{0} |S_{j}|} \, e^{8 \Vert \Omega \Vert _{\lambda }} \, \le \, 4 \, \Omega _{j} e^{\lambda j} e^{8 \Vert \Omega \Vert _{\lambda }} e^{(8 \Omega _{0}-\lambda )j} \\ \Vert \Gamma _{G}^{s}(U_{j}) - \Gamma _{G_{S_{k}}}^{s}(U_{j}) \Vert \,&\le \, \Vert U_{j}\Vert \, e^{2 \Omega _{0} |S_{j}|} \, e^{8 \Vert \Omega \Vert _{\lambda }} e^{(8 \Omega _{0} - \lambda ) (k-j)},\\&\le \, 4 \, \Omega _{j} \, e^{8 \Omega _{0} j} \, e^{8 \Vert \Omega \Vert _{\lambda }} \, e^{(8 \Omega _{0} - \lambda ) (k-j)}\, \\&= \, 4 \, \Omega _{j} \, e^{\lambda j} \, e^{8\Vert \Omega \Vert _{\lambda }} \, e^{(8\Omega _{0} - \lambda )k}. \end{aligned}$$

Next, using (58) and the previous estimates, we deduce for every $k \ge 1$ and $|s| \le 1$ that

$$\begin{aligned} \Vert \Gamma _{G}^{s}(U) \Vert \, , \, \Vert \Gamma _{G_{S_{k}}}^{s}(U) \Vert \,&\le \, \sum _{j \ge 1} 4 \, \Omega _{j} \, e^{\lambda j} \, e^{8\Vert \Omega \Vert _{\lambda }} \, \le \, 4 \, \Vert \Omega \Vert _{\lambda } \, e^{8 \Vert \Omega \Vert _{\lambda }} \, \le \, e^{12 \Vert \Omega \Vert _{\lambda }}, \end{aligned}$$

and also that

$$\begin{aligned} \Vert \Gamma _{G}^{s}(U) - \Gamma _{G_{S_{k}}}^{s}(U_{S_{k}}) \Vert&\le \, \sum _{j=1}^{k}{\Vert \Gamma _{G}^{s}(U_{j}) - \Gamma _{G_{S_{k}}}^{s}(U_{j}) \Vert } + \sum _{j>k}\Vert \Gamma _{G}^{s}(U_{j}) \Vert \\&\le \, 4 \, e^{8\Vert \Omega \Vert _{\lambda }} \, e^{(8 \Omega _{0} - \lambda )k} \,\sum _{j=1}^{k} \Omega _{j} e^{\lambda j} + 4 \, e^{8 \Vert \Omega \Vert _{\lambda }} \, \sum _{j>k} \Omega _{j} e^{\lambda j} \, e^{(8 \Omega _{0}-\lambda )j}\\&\le \, 4 \, \Vert \Omega \Vert _{\lambda } \, e^{8\Vert \Omega \Vert _{\lambda }}\, e^{(8 \Omega _{0} - \lambda )k}\\&\le \, e^{12 \Vert \Omega \Vert _{\lambda }}\, e^{(8 \Omega _{0} - \lambda )k} . \end{aligned}$$

Applying these estimates to (57) and reasoning as in the proof of Theorem 3.1 we conclude the result. $\square$

#### Theorem 5.6

Let us assume that the boundary Hamiltonians $(G_{\partial \mathcal {R}})_{\mathcal {R}}$ have a local decomposition $(g_{\partial \mathcal R}^{X})$ with exponential decay $\Vert \Omega \Vert _{\lambda } < \infty$ for some $\lambda > 8 \Omega _{0}$, and $\eta$-homogeneous for an absolutely summable sequence $\eta (\ell )$. Then, for every pair of adjacent rectangles *AB* as above and every $\ell \ge 1$ the elements

$$\begin{aligned} G := \sum _{X \subset (\partial A {\setminus } \partial B) {\setminus } S_{\ell }}{g_{X}^{\partial AB}}\quad \quad \text{ and } \quad \quad \widetilde{G} := \sum _{X \subset (\partial A {\setminus } \partial B) {\setminus } S_{\ell }}{g_{X}^{\partial A}} \end{aligned}$$

satisfy

$$\begin{aligned} \Vert e^{G}e^{-\widetilde{G}} - \mathbb {1}\Vert \le \exp {\big [ e^{16 \Vert \Omega \Vert _{\lambda }} {\sum }_{j> \ell }\, \eta (j) \big ]} e^{16 \Vert \Omega \Vert _{\lambda }} \Big ( {\sum }_{j > \ell }\, \eta (j)\Big ). \end{aligned}$$

#### Proof

Let us denote by $\mathcal {F}$ the family of all sets $X \subset \partial A {\setminus } \partial B$. We are going to split $\mathcal {F}$ into the following subfamilies: for each $1 \le j \le k$ let us define inductively

$$\begin{aligned} \mathcal {F}_{j,k}:= \{ X \in \mathcal {F} :X \subset S_{k}, X \cap S_{j} \ne \emptyset \} {\setminus } \bigcup \{ \mathcal {F}_{j',k'} :j'< j \text{ or } k' < k \}. \end{aligned}$$

This ensures that every set *X* in $\mathcal {F}_{j,k}$ has support contained in $S_{j,k}:=S_{k} {\setminus } S_{j-1}$ ($S_{0}:=\emptyset$) and diameter greater than or equal to $k-j$, since it intersects both $S_{k} {\setminus } S_{k-1}$ and $S_{j} {\setminus } S_{j-1}$. As a consequence we can estimate the norm of

$$\begin{aligned} U_{j,k}:= \sum _{X \in \mathcal {F}_{j,k}}{g_{X}^{\partial AB} - g_{X}^{\partial A}}\, \end{aligned}$$

using the $\eta$-homogeneity condition by

$$\begin{aligned} \Vert U_{j,k}\Vert \le \sum _{x \in S_{k} {\setminus } S_{k-1}} \sum _{X \ni x} \Vert g_{X}^{\partial AB} - g_{X}^{\partial A} \Vert \le 8 \eta (j) \Omega _{k-j}. \end{aligned}$$

Applying the locality estimates from Lemma 5.4 to $U_{j,k}$, we further get that for $|s| \le 1$

$$\begin{aligned} \Vert \Gamma _{G}^{s}(U_{j,k})\Vert \, \le \, \Vert U_{j,k}\Vert \, e^{2 \Omega _{0} |S_{j,k}|} \, e^{8 \Vert \Omega \Vert _{\lambda }}\, \le \, 8 \eta (j) \, \Omega _{k-j} \, e^{4 \Omega _{0}(k-j)} \, e^{8 \Vert \Omega \Vert _{\lambda }}. \end{aligned}$$

Therefore, using that the perturbation can be written as

$$\begin{aligned} U := G - \widetilde{G} = \sum _{\ell < j \le k}{U_{j,k}} \end{aligned}$$

we conclude that for $|s| \le 1$

$$\begin{aligned} \Vert \Gamma ^{s}_{G}(U)\Vert \le \sum _{\ell < j \le k} \Vert \Gamma ^{s}_{G}(U)\Vert \,&\le \, 8 e^{8\Vert \Omega \Vert _{\lambda }} \sum _{j> \ell } \eta (j) \, \sum _{k \ge j} \Omega _{k-j} e^{4 \Omega _{0}(k-j)}\\&\le \, 8 e^{8\Vert \Omega \Vert _{\lambda }} \Vert \Omega \Vert _{\lambda } \sum _{j> \ell }{\eta (j)}\\&\le \, e^{16 \Vert \Omega \Vert _{\lambda }} \sum _{j>\ell }{\eta (j)}. \end{aligned}$$

Applying these estimates to (57) and reasoning as in the proof of Theorem 3.1 we conclude the result. $\square$

### Main result

#### Theorem 5.7

Let $(\rho _{\partial \mathcal {R}})_{\mathcal {R}}$ be a family of boundary states whose boundary Hamiltonians $(G_{\partial \mathcal {R}})_{\mathcal {R}}$ have a local decomposition with exponential decay $\Vert \Omega \Vert _{\lambda }< \infty$ for some $\lambda > 8 \Omega _{0}$, and also $\eta$-homogeneous for an absolutely summable sequence $\eta (\ell )$ with sum $\Vert \eta \Vert$. Then, $(\rho _{\partial \mathcal {R}})_{\mathcal {R}}$ is approximately factorizable with function

$$\begin{aligned} \varepsilon (\ell ) = \exp \big [ e^{K\, (1 + \Vert \Omega \Vert _{\lambda } + \Vert \eta \Vert )} \big ] \, \big ( \, e^{(8 \Omega _{0} - \lambda ) \ell } \, + {\sum }_{k > \ell } \,\, \eta (k) \, \big ) \end{aligned}$$

for an absolute constant $K > 0$.

#### Proof

Let us fix $\ell >0$ and consider an $\ell$-admissible triplet of rectangles *ABC* as in Fig. 3. For simplicity let us denote the corresponding boundary Hamiltonians of *ABC*, *AB*, *BC* and *B* respectively as

$$\begin{aligned} \rho _{ABC} = e^{2 Q_{azc}}, \,\,\, \rho _{AB} = e^{2 R_{a z \gamma }}, \,\,\, \rho _{BC} = e^{2 S_{\alpha z c}}, \,\,\, \rho _{B} = e^{2 T_{\alpha z \gamma }}. \end{aligned}$$

Following the same idea from [22] one considers the following invertible matrices

$$\begin{aligned} \Delta _{az}&:= e^{Q_{ax}} e^{-Q_{y}} e^{Q_{axy}}, \quad \Upsilon _{\alpha z}:= e^{T_{\alpha x}} e^{-T_{y}} e^{T_{\alpha xy}},\\ \Delta _{zc}&:= e^{Q_{xyc}} e^{-Q_{x}} e^{Q_{yc}}, \quad \Upsilon _{z \gamma } :=e^{T_{xy\gamma }} e^{-T_{x}} e^{T_{y\gamma }}, \end{aligned}$$

and shows that they provide the desired approximate factorization. We are going to explicitly check this for $\rho _{AB}$ and $\sigma _{AB}:=\Upsilon _{z\beta }\Delta _{az}$, namely that

$$\begin{aligned} \Vert \rho _{AB}^{1/2} \, \sigma _{AB}^{-1} \, \rho _{AB}^{1/2} - \mathbb {1} \Vert < \varepsilon (\ell )\, \end{aligned}$$

for a suitable function $\varepsilon (\ell )$ as above. We can explicitly write

$$\begin{aligned} \rho _{AB}^{1/2} \, \sigma _{AB}^{-1} \, \rho _{AB}^{1/2} = \left( e^{R_{axy\gamma }} e^{-Q_{axy}} e^{Q_{y}} e^{-T_{y \gamma }}\right) \left( e^{-Q_{ax}} e^{T_{x}} e^{-T_{xy\gamma }} e^{R_{axy\gamma }} \right) . \end{aligned}$$

Let us show that each of the two factors can be suitably approximated by the identity. We deal with the first factor, the second one is analogous. Let us decompose

$$\begin{aligned} \begin{aligned} e^{R_{axy\gamma }} e^{-Q_{axy}} e^{Q_{y}} e^{-T_{y\gamma }} \, = \,&\big ( e^{R_{axy\gamma }} e^{-R_{axy} - R_{\gamma }} \big ) \, \big ( e^{R_{axy}} e^{-Q_{axy}} \big )\\&\big (e^{R_{\gamma }} e^{-T_{\gamma }}\big ) \, \big (e^{Q_{y}}e^{-T_{y}}\big ) \, \big (e^{T_{y} +T_{\gamma }} e^{-T_{y \gamma }}\big ). \end{aligned} \end{aligned}$$

By Theorems 5.5 and 5.6 we know that there is an absolute constant $K > 0$ such that the five factors are uniformly bounded by

$$\begin{aligned} \exp {\big (\, e^{K( 1+ \Vert \eta \Vert + \Vert \Omega \Vert _{\lambda })} \, \big )}, \end{aligned}$$

and moreover we can approximate

$$\begin{aligned}&e^{R_{axy\gamma }} e^{-R_{axy} - R_{\gamma }} \,\, \approx \,\, e^{R_{y\gamma }} e^{-R_{y} - R_{\gamma }}\\&e^{R_{\gamma }} e^{-T_{\gamma }}\,,\,\, e^{Q_{y}}e^{-T_{y}} \,,\,\, e^{R_{axy}} e^{-Q_{axy}} \,,\,\, e^{R_{y}} e^{-T_{y}}\,,\,\,e^{R_{y \gamma }} e^{-T_{y \gamma }} \,\, \approx \,\, \mathbb {1} \end{aligned}$$

with an error of order

59 $$\begin{aligned} \exp \big [ e^{K(1 + \Vert \Omega \Vert _{\lambda } + \Vert \eta \Vert )} \big ] \, \big ( \, e^{(8 \Omega _{0} - \lambda ) \ell } \, + {\sum }_{k > \ell } \,\, \eta (k) \, \big ). \end{aligned}$$

Thus, applying these estimates to the above decomposition we conclude that

$$\begin{aligned} e^{R_{axy\gamma }} e^{-Q_{axy}} e^{Q_{y}} e^{-T_{yc}} \,&\approx \big ( e^{R_{y\gamma }}e^{-R_{y} - R_{\gamma }} \big ) \, \big ( e^{R_{y}} e^{-T_{y}}\big ) \big ( e^{R_{\gamma }} e^{- T_{\gamma }} \big ) \, \big ( e^{T_{y} + T_{\gamma }} e^{-T_{y\gamma }} \big )\\&= e^{R_{y \gamma }} e^{-T_{y \gamma }} \approx \mathbb {1}, \end{aligned}$$

with an accumulated error of the same type (59) for a suitable absolute constant *K*. $\square$

## Final Remarks and Conclusions

We have presented locality estimates for complex time evolution of local observables in one-dimensional systems with interactions decaying exponentially fast, applying them to extend previous works on the clustering property of KMS states and the spectral gap problem for parent Hamiltonians of PEPS.

More specifically, we have shown that if the interactions on the infinite one-dimensional lattice decay as $\Omega _{n} = O(e^{- \lambda n})$ for some $\lambda > 0$, then for every local observable *Q* the infinite-volume time evolution operator $s \longmapsto \Gamma ^{s}(Q)$ is well-defined, analytic and quasi-local (with exponential tails) on the strip $|{\text {Im}}(s)| < \lambda /(4 \Omega _{0})$. In particular, for interactions that decay faster than any exponential we get analyticity on the whole complex plane, recovering Araki’s result for finite-range interactions. As far as we know, there is no 1D model exhibiting this type of threshold, although there are examples in 2D of finite range interactions with this property [6].

We have also shown that, under the previous conditions, the infinite-volume KMS state at inverse temperature $\beta$ has exponential decay of correlations whether $0< \beta < \lambda /(2 \Omega _{0})$. This leaves open the existence of phase transitions at lower temperatures, which might be unexpected according to the folklore statement that 1D systems with short range Hamiltonians do not exhibit phase transitions. A similar constraint in terms of $\lambda$ and $\Omega _{0}$ also appears in the result for PEPS.

In both applications, these seeming thresholds arise from combining the Araki–Dyson expansional formulas for local perturbations with the prior locality estimates. Let us remark that there is an alternative perturbation formula due to Hastings [17] providing a factorization $e^{-(H+U)} = O \, e^{-H} \, O^{\dagger }$, where now the locality properties of *O* depend on the locality of the perturbation *U* via the real-time Lieb–Robinson bounds. Thus, the locality estimates for *O* are better than those of Araki’s expansional $E=e^{-\frac{1}{2}(H+U)}e^{\frac{1}{2} H}$. However, it is not clear how to remake the argument to prove exponential decay of correlations replacing *E* with *O*, leaving this possibility open.

## Footnotes

1 We are following the definition given in [22]. See there for a comparison with the definition given in [9].
